# Enhanced performance of nanoplate-carbon nanotube reinforced poly(butylene succinate) nanocomposites for sustainable packaging and multifunctional applications

**DOI:** 10.1016/j.fochx.2025.102989

**Published:** 2025-09-09

**Authors:** Chi-Hui Tsou, Xin Huang, Fei-Fan Ge, Jarrn-Horng Lin, Pranut Potiyaraj, Charasphat Preuksarattanawut, Tao Yang, Xue-Fei Hu

**Affiliations:** a Material Corrosion and Protection Key Laboratory of Sichuan Province, School of Materials Science and Engineering, Sichuan University of Science and Engineering, Zigong 643000, China; b Department of Petrochemistry and Polymer Science, Faculty of Science, Chulalongkorn University, Bangkok 10330, Thailand; c Department of Metallurgical Engineering, Faculty of Engineering, Chulalongkorn University, Bangkok 10330, Thailand; d Department of Material Science, National University of Tainan, Tainan, 70005, Taiwan, ROC; e Sichuan Zhixiangyi Technology Co., Ltd, Sichuan Bozhiduo Technology Co., Ltd., Chengdu 610051, China; f Zigong Zhishengxin Technology Co., Ltd., Zigong 643000, China

**Keywords:** Modified poly(butylene succinate) (MPBS), ZnO nanoplate decorated carbon nanotubes (CNT-ZNP), Fruit preservation, Barrier properties, Food packaging applications

## Abstract

This study developed modified poly (butylene succinate) (MPBS) nanocomposites reinforced with ZnO nanoplate-decorated carbon nanotubes (CNT-ZNP) to improve mechanical, thermal, and functional properties for food packaging applications. MPBS was synthesized via grafting of maleic anhydride to enhance compatibility with nanofillers. Incorporation of only 0.1 g/hg CNT-ZNP led to substantial improvements in tensile strength (36.5 %), elongation at break (108.6 %), and yield strength (41.6 %), demonstrating that enhanced performance can be achieved with minimal nanofiller content. The nanocomposite also exhibited enhanced crystallinity, thermal stability, and water vapor barrier performance. Food preservation tests demonstrated that MPBS/CNT-ZNP films effectively maintained banana freshness over 14 days and inhibited microbial growth in raw chicken for up to 108 h. These outcomes highlight the material's potential to extend shelf life and reduce spoilage in perishable products. Overall, the MPBS/CNT-ZNP nanocomposites combine mechanical robustness with antimicrobial and preservation functionality, supporting their application as sustainable and multifunctional food packaging films.

## Highlights

• 0.1 g/hg CNT-ZNP improves MPBS mechanical properties and crystallinity.
• ZnO nanoplate-CNT reinforcement boosts thermal stability and barrier properties.
• Low CNT-ZNP content offers cost-effective benefits for nanocomposites.
• MPBS/CNT-ZNP films show strong antibacterial effects for food packaging.
• Potential for effective preservation of fresh produce and raw meat.

## Graphical abstract

**Figure:**
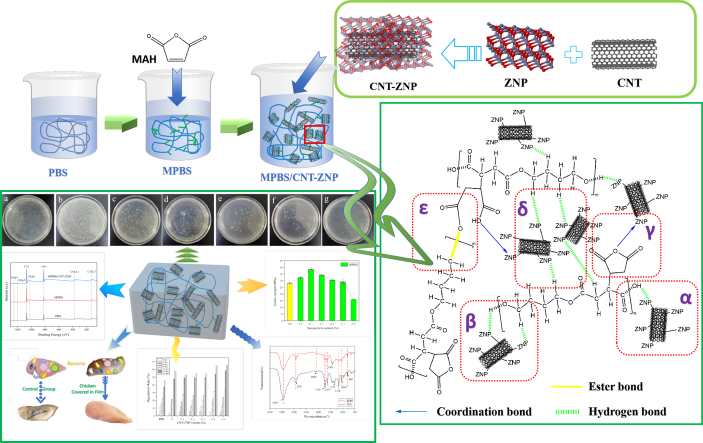

## 1 Introduction

The increasing concentration of carbon dioxide due to the use of fossil fuels and the increasing production of non-degradable polymer composites has a serious impact on global warming and climate change (Li et al., 2015). In recent years, research on environment-friendly polymer composites with renewable resources has attracted more attention (Faria et al., 2020; Kalia et al., 2011). Natural materials and clean, easy to use, harmless and environmentally friendly materials used to manufacture various functional materials have gradually replaced traditional materials (Mohammad et al., 2020). In the field of packaging industry, the application of polymer products has been rapidly increasing (Zhong et al., 2020). Because of the suitability, security, and low price of polymers, there has been a rapid increase. The proportion of current plastics used for packaging accounts for 41 g/hg of the total plastic mass (Kraśniewska et al., 2020) [6]. The following categories are commonly used commercially: polyvinyl chloride (Belukhichev et al., 2020) (PVC), polystyrene (Kelly et al., 2020) (PS), Polypropylene (Peng et al., 2019) (PP), polyethylene (Kirsh et al., 2020) (PE), etc. The aforementioned packaging materials, derived from petroleum-based polymers, are typically disposed of in natural settings where they do not biodegrade, thereby causing environmental damage (Bari et al., 2016).

Polybutylene succinate (PBS) was selected as the base polymer in this study due to its excellent biodegradability (Narancic et al., 2018; Puchalski et al., 2018), moderate thermal stability, and favorable mechanical performance (Huang et al., 2018a, Huang et al., 2018b; Kuang et al., 2018; Yao et al., 2017). Compared to other biodegradable polymers such as polylactic acid (PLA), PBS also offers better toughness and processability, making it a practical choice for scalable and sustainable food packaging applications. However, pure PBS still has certain limitations, including relatively high production costs and suboptimal functional properties, which restrict its broader commercial adoption (Kopitzky et al., 2019; Liminana et al., 2018; Huang et al., 2018a, Huang et al., 2018b; Sharma et al., 2020). To overcome these drawbacks, various nanomaterials have been introduced to enhance PBS performance. The incorporation of nanofillers has been shown to improve its antibacterial activity (Chiu et al., 2017; Ezzeddine et al., 2021; Gowman et al., 2018; Hazhir et al., 2019; Li et al., 2017; Marek et al., 2020; Sommai et al., 2021; Wang et al., 2021; Zhang et al., 2018), tensile strength (Azhar et al., 2019; Lule et al., 2020; Qi et al., 2019), electrical conductivity (Al Mulla et al., 2017; Kaur et al., 2017; Zelalem et al., 2018), and barrier properties (Ferreira et al., 2017; Gonghua et al., 2018; Tsou et al., 2020). As a result, PBS-based nanocomposites have attracted increasing attention as promising materials for multifunctional and eco-friendly packaging solutions (Alebooyeh et al., 2013; Lin et al., 2011).

Carbon nanotubes (CNT) (Ürk et al., 2016; Volynets et al., 2017) are good enhancers used in polymer matrices and can be applied as nano additives with excellent properties (Zhao et al., 2008). They have excellent electrical conductivity (Al-Saleh et al., 2009; Ma et al., 2008). But CNT nanomaterials do not exhibit good antibacterial properties. However, nano Zn has excellent antibacterial properties, which can make the target material a certain degree of antibacterial (Tsou et al., 2016; Tsou et al., 2017; Jones et al., 2008; Zhang et al., 2018). So, combining CNT with Zn to prepare ZnP decorated multi walled carbon nanotubes (CNT-ZNP) could not only enhance the comprehensive performance of MPBS (Spencer et al., 2017; Sun et al., 2021), but also endow the nanocomposites with antibacterial properties (Shrestha et al., 2017). For example, a polyvinyl alcohol nanocomposite membrane modified with CNT and Zn exhibited good tensile strength and water resistance, along with improved hydrophobicity (Sun et al., 2021). CNTs doped with ZnO were also used to reinforce polyvinyl alcohol, resulting in excellent tensile and antibacterial properties, as well as an extended shelf life of chicken (Wen et al., 2022). In another study, CNTs doped with ZnO were incorporated as nanofillers into polyphenylene sulfide, which significantly enhanced its tensile strength, water resistance, and antibacterial activity (Ge et al., 2022). Abinaya et al. have demonstrated that ZnO nanoplate (ZNP) possesses excellent antimicrobial properties (Abinaya et al., 2016). Moreover, due to its planar structure, ZNP might offer enhanced barrier performance (Malik et al., 2021). Abdullah and his team also indicated that the incorporation of ZNP can improve the water vapor permeability of polymers (Abdullah et al., 2022). Consequently, Chen and colleagues utilized ZNP-modified graphene (G-ZNP) to enhance the tensile and thermal properties of polyvinyl alcohol hydrogel. They also demonstrated that G-ZNP can impart antimicrobial characteristics to the polyvinyl alcohol hydrogel (Chen et al., 2023).

The synthesis of zinc nanoparticle (ZNP)-modified carbon nanotube (CNT) nanomaterials was undertaken in this study (CNT-ZNP) for their potential application as efficient nanofillers in polymer films. A commercially available PBS sample (TH803S) was selected based on its availability and mechanical properties representative of materials commonly used in biodegradable packaging. The fundamental characteristics of TH803S, along with those of two others widely studied commercial PBS grades, are summarized in Table S1 (Bing et al., 2024; Ostrowska et al., 2019; Phua et al., 2013; Xie et al., 2014). These data provide a comparative basis for evaluating the material's suitability in the context of mechanical performance, processability, and application potential in sustainable packaging systems.

Bananas and chicken meat were selected as representative food models to evaluate the real-world applicability of the nanocomposite films. Bananas are climacteric fruits with high ethylene sensitivity and rapid spoilage rates, making them widely used in fruit preservation research to assess barrier performance and microbial inhibition under ambient conditions. Chicken breast, due to its high moisture and protein content, is highly perishable and serves as an effective model for evaluating antimicrobial efficacy in meat packaging.

Based on these considerations, the specific objectives of this study were set as follows: (1) PBS-TH803S was investigated as a representative biodegradable polymer matrix for sustainable packaging applications; (2) The interfacial compatibility was enhanced and functional performance of PBS through maleic anhydride grafting, yielding modified PBS (MPBS); (3) ZnO-coated CNT was incorporated carbon nanotubes (CNT-ZNP) into MPBS to improve its mechanical, thermal, barrier, antibacterial, and anti-inflammatory properties; and (4) The food preservation performance of MPBS/CNT-ZNP nanocomposite films was evaluated using bananas and chicken as model systems, by monitoring weight loss, microbial growth, color change, total soluble solids, and pH during storage. The ultimate goal is to develop multifunctional, biodegradable nanocomposites with enhanced preservation efficacy and broad application potential in packaging of meat, fruits, and other moisture-sensitive foods.

## 2 Experimental

### 2.1 Materials

Poly(butylene succinate) (PBS, model TH803S) was supplied by Xinjiang Lanshan Tunhe Co., Ltd., Urumqi, Xinjiang, China. Maleic anhydride (MAH, ≥99.0 % purity, analytical grade) was purchased from Shanghai Titan Scientific Co., Ltd., Shanghai, China. Dicumyl peroxide (DCP, 98 %, CAS No. 80–43-3) was obtained from Kandis Chemical Co., Ltd., Wuhan, Hubei, China. Sodium chloride (NaCl, analytical reagent grade) was provided by Chengdu Kelong Chemical Reagent Co., Ltd., Chengdu, Sichuan, China. Beef extract, agar powder, and peptone (all microbiological grade) were obtained from Beijing Oboxing Biotechnology Co., Ltd., Beijing, China.

### 2.2 Preparation of CNT-ZNP

ZNP was obtained from Sichuan Zhixiangyi Technology Co., Ltd. (Sichuan, China). ZNP was coated on the CNT surface by means of a wetting dispersion method. The ratio of CNT to ZNP was 1:2. The CNTs were dispersed in ethanol using a probe-type ultrasonic processor (FS-1088 N, Sxonic Instruments, Shanghai, China) for 45 min to ensure homogeneity. Then, ZNP was added to the CNT-ethanol mixture solution, which was then ultrasonicated for another 1.5 h to disperse the ZNP well and avoid its aggregation. Afterward, the mixture was filtered to separate ethanol. To completely remove the residual ethanol, the mixture was placed in an oven at 65 °C for 3 h, and it was dried for another 1 h at a higher temperature of 120 °C.

Its appearance can be observed from SEM and TEM, as shown in Fig. S1a and S1b. According to the information provided by the manufacturer, ZNP is a sheet with a thickness of 20 nm. From the SEM images in Fig. S1a, it is also speculated that the flake nanomaterials are sheet-like ZNP. Because ZNP is coated outside the CNT, it cannot be found in SEM image. For the TEM, it is also not easy to observe. After many shots from TEM, it can be observed from TEM images that a single layer of thin ZNP is attached to the CNT (see Fig. S1b).

### 2.3 Preparation of MPBS and MPBS/CNT-ZNP nanocomposites

MPBS was synthesized via a three-step melt grafting process (Tsou et al., 2015). PBS pellets were first mixed with 0.4 g/hg dicumyl peroxide (DCP) for 5 min, followed by the addition of 4 g/hg maleic anhydride (MAH) and continued mixing for 3 min to form PBS-g-MAH. The grafting reaction was carried out at 125 °C and 60 rpm in an internal mixer. The resulting product was dissolved in toluene at 110 °C to form a clear solution, precipitated in methanol, filtered, and thoroughly washed to remove residual DCP and MAH. The precipitate was then vacuum-dried at 80 °C for 8 h using a vacuum oven (model DZF-6050, Shanghai Jinghong Laboratory Instrument Co., Ltd., Shanghai, China). The MPBS matrix was prepared by blending PBS-g-MAH (10 g/hg) with PBS (90 g/hg). Based on preliminary optimization, CNT-ZNP nanofillers were introduced in varying amounts (0–0.5 g/hg), as detailed in Table S2. Unless otherwise specified, all materials were vacuum-dried at 82 °C for 7.5 h using the same vacuum oven before processing.

The blending of MPBS with CNT-ZNP was performed using a torque rheometer at 125 °C: first at 100 rpm for 3 min, then at 200 rpm for another 3 min. The obtained composites were hot-pressed at 125 °C and 15 MPa for 10 min using a manual hot press and allowed to cool naturally to room temperature. Dumbbell-shaped specimens were cut using a custom mold and stored in moisture-proof containers for subsequent characterization. The overall fabrication procedure of the MPBS/CNT-ZNP nanocomposite films is schematically depicted in Fig. 2.

## 3 Testing and characterization

### 3.1 Fourier transform infrared spectroscopy (FTIR) for PBS and MPBS

FTIR spectra were recorded using a Fourier transform infrared spectrometer (Nicolet 6700, Thermo Fisher Scientific, Waltham, MA, USA) to identify the functional groups present in the nanocomposites. Prior to measurement, samples were conditioned in a vacuum oven at 80 °C for 4 h. The PBS and MPBS were then ground into powder using a mortar and pestle. This powder was mixed with potassium bromide and pressed into a disc shape in a mold under a pressure of 15 MPa. The prepared samples were placed and securely fixed in the sample chamber. A background scan was conducted before each sample analysis to ensure the accuracy of the test data. The experimental range spanned from 4000 to 500 cm^−1^, with an average of 8 scans per sample and a resolution set at 4 cm^−1^, resulting in the acquisition of the FTIR spectra of the samples.

### 3.2 X-ray photoelectron spectroscopy for PBS and MPBS

X-ray photoelectron spectroscopy (XPS) was conducted using a K-Alpha^+^ spectrometer (Thermo Fisher Scientific, Waltham, MA, USA) to analyze elemental distribution, surface functional groups, and the carbon atom hybridization states of the samples. The powdered sample was placed within a testing environment with a background vacuum of less than 10–8 mbar (*P* < 10–8 mbar). An Al K X-ray source with a working energy of 100 Wα (1486.6 eV) was used for scanning and analysis, employing 50 eV setting. All binding energies were calibrated using the carbon (C1s) and oxygen (O1s) peak, respectively (about 284.8 and 531.8 eV).

### 3.3 Attenuated Total reflectance (ATR) for MPBS/CNT-ZNP

The prepared samples were first cropped into square flakes of 10 mm × 10 mm, put into a vacuum drying box at 80 °C for 8 h. The Nicolet 6700 FTIR spectrometer, operating in transmission mode, was used to measure the infrared spectra of the specimens across a wavelength range of 4000 to 500 cm^−1^. To characterize the IR spectra of samples, the instrument averaged 32 scans at a resolution of 2 cm^−1^ to clearly identify the peaks.

### 3.4 Gel permeation chromatography (GPC)

Gel permeation chromatography (GPC) experiments were carried out using a Waters 1515 chromatographic system (Waters Corporation, Milford, MA, USA), equipped with a MIXED 7.5 × 50 mm PL guard column and two MIXED-C 7.5 × 300 mm columns. This setup also featured a differential refractive index detector. The elution was carried out using HPLC-grade chloroform as the solvent, under a steady temperature of 35 °C and a flow rate set at 1 mL/min.

### 3.5 Mechanical performance test

To assess the mechanical strength of the MPBS/CNT-ZNP nanocomposite films, a computerized universal testing machine (model FBS-10KNW, Shenzhen Kexin Instrument Co., Ltd., Shenzhen, China) was used. Initially, standard type 5 test pieces, each 50 mm long with a gauge distance of 25 mm, were prepared according to the GB/T 1040–2006 standard. Measurements of width and thickness at four different points within the test length were taken and averaged. For each sample, five tensile test specimens were prepared. The tensile load was applied at a rate of 2 mm/min until the specimens broke. The average of the five tensile test results for each sample was used to determine the tensile strength and elongation at break of the nanocomposites.

### 3.6 Morphological characterization

To investigate the fracture morphology of the nanocomposites, a scanning electron microscope (VEGA 3SBU, TESCAN, Brno, Czech Republic) was used. The SEM micrographs represent the tensile fracture cross-sections of the composite films, providing detailed visualization of the morphology and distribution of CNT-ZNP particles within the MPBS matrix. Prior to imaging, the sample surfaces were coated with a thin layer of gold using a sputter coater operating at 15 kV and 8 mA for 30 s to enhance conductivity and image clarity. After coating, the specimens were placed in the microscope's observation chamber, and high-magnification images (1000×) were acquired at an accelerating voltage of 20 kV to analyze the surface and internal structures of the films.

### 3.7 EDS test

Elemental composition and distribution within the samples were analyzed using an energy-dispersive X-ray spectroscopy (EDS) system (Bruker, Berlin, Germany) integrated into the scanning electron microscope (VEGA 3SBU, TESCAN, Brno, Czech Republic). For the EDS measurements, the machine was operated at an accelerating voltage of 15 kV, and the samples were positioned at a working distance of 15 mm from the detector. Given that zinc (Zn) is present in CNT-ZNP but not in the PBS matrix, the elemental mapping analysis specifically targeted the distribution of zinc within the nanocomposites. Each sample was scanned three times in a flat and uniform manner to ensure reproducibility and accuracy. The EDS mapping allowed us to visualize the spatial distribution and concentration of zinc, providing insights into the dispersion of CNT-ZNP within the PBS matrix.

### 3.8 Comprehensive thermal analysis

A differential scanning calorimeter (model DSC 200 F3, NETZSCH, Selb, Germany) was employed to determine the crystallization temperature and degree of crystallinity of the composite materials. Each sample used for the test weighed approximately 8 mg. The testing protocol involved heating the sample from ambient temperature to 150 °C at a constant rate of 10 °C/min. Upon reaching 150 °C, the temperature was held for 10 min before allowing the sample to cool naturally to room temperature. The sample was then reheated to 150 °C at the same rate to collect thermal data. The crystallinity (*Xc*) of the materials was calculated using the following equation:

(1) $$X_{c}=\frac{∆H_{m}}{\left(1-a\right)∆H_{m}^{0}}\times 100\%$$

In this formula, “α” represents the percentage of filler content in the material. “*∆H*_*m*_” is the measured enthalpy of fusion, expressed in Joules per gram (J/g), and “*∆H*_*m*_^*0*^” is the theoretical enthalpy of PBS (polybutylene succinate) at full (100%) crystallization, which is 200 J/g. This calculation helps us understand how much of the material forms a crystalline structure under these conditions.

### 3.9 X-ray diffraction characterization

The crystalline structure of the composite films was analyzed using an X-ray diffractometer (model D2 PHASER, Bruker, Karlsruhe, Germany). Characteristic diffraction patterns indicative of the internal structure was recorded. The scans were performed over a wide-angle range from 5° to 60° (2θ), with a step size of 0.02° and a measurement time of 0.2 s per step. This scanning protocol enabled the acquisition of a comprehensive set of diffraction data for the MPBS/CNT-ZNP composite materials.

### 3.10 Thermal stability test

Thermogravimetric analysis (TGA) was performed using a thermogravimetric analyzer (model STA 409PC, NETZSCH, Selb, Germany) after the samples were dried at 80 °C for 4 h. Each sample, weighing 8–10 mg, was placed in a crucible and analyzed under a nitrogen atmosphere. The test protocol involved heating from room temperature to 650 °C at a rate of 10 °C/min, followed by natural cooling. Three replicates were tested for each group, and the average results were used to generate thermogravimetric (TG) and derivative thermogravimetric (DTG) curves.

### 3.11 Water vapor barrier test

The water vapor barrier properties of the composite PBS and MPBS/CNT-ZNP materials were evaluated using a water vapor transmission rate test system (model W3/060, Jinan Languang Electromechanical Technology Co., Ltd., Jinan, China). Prior to testing, the nanocomposite films were cut into circular specimens with a diameter of 75 mm and a thickness of 0.05 mm, and then dried in an oven at 80 °C for 8 h. The specimens were subsequently placed in the test chamber, which contained a small amount of distilled water. The internal temperature of the chamber was maintained at 26 °C, and the measurements were conducted at 30-min intervals. Relative humidity in the chamber was controlled at 90 %, and a fixed humidity gradient was achieved with 10-min intervals between measurements for a total of 10 cycles.

The test results were reported as water vapor permeability (WVP), expressed in grams per square centimeter per second per Pascal (g·cm/cm^2^·s·Pa). The WVP coefficient was calculated as the average of four specimens. The formula used for calculating WVP is shown below:

(2) $$P_{v}=\frac{∆m∙d}{A∙T∙∆P}$$

Where Pv = water vapor permeability,

∆m = mass increment in t time, g;

d = sample thickness, cm;

A = water vapor permeable area of sample, cm^2^;

T = time interval after mass increment stabilized, s;

∆P = water vapor pressure difference across sample, Pa.

### 3.12 Water absorption test

The water absorption test was conducted on PBS, MPBS, and MPBS/CNT-ZNP nanocomposites, with five specimens prepared for each material to ensure accuracy and reliability. The average water absorption value was calculated from these five measurements. Each specimen (10 × 10 × 1 mm) was first dried in an oven at 80 °C for 8 h, then weighed to record its initial mass. The specimens were subsequently immersed in water at an ambient temperature of 25 °C for 24 and 48 h. After immersion, they were carefully removed, excess water was blotted off, and they were reweighed. The water absorption was calculated using the following equation:

(3) $$M=\frac{M_{1}-M_{2}}{M_{2}}\times 100\%$$

Where M = water uptake;

M_1_ = weight of the wet specimen.

M_2_ = original weight of the dry specimen.

### 3.13 Contact angle test

The hydrophilic properties of the nanocomposites were evaluated using a contact angle tester (model JC2000D, Shanghai Zhongchen Digital Technology Equipment Co., Ltd., Shanghai, China). A droplet of 2 μL distilled water was vertically deposited onto the surface of each sample. The contact angle was measured three times per specimen, and the average value was calculated to ensure consistency. The parameters included a film thickness of 1 mm, with measurements taken at time intervals of 0, 15, and 30 s.

### 3.14 Biodegradability assessment

Biodegradation of PBS and MPBS/CNT-ZNP nanocomposites was assessed using a soil burial test. For each composite, five film specimens (10 mm × 10 mm) were prepared and their initial masses recorded using an electronic balance. The samples were then buried in soil maintained at a constant temperature and humidity to simulate natural environmental conditions. Evaluations were conducted every 30 days over a 180-day period. After each interval, the specimens were removed, rinsed with deionized water to remove residual soil, and oven-dried at 105 °C for 4 h. Each test was performed in triplicate, and the average weight loss was used to assess the extent of biodegradation.

### 3.15 Antibacterial evaluation

Quantitative antibacterial assessments were performed (Tsou & Guo et al., 2020) to evaluate the efficacy of pure PBS, MPBS and MPBS/CNT-ZNP composites in inhibiting *E. coli*. The bacteria used in this study, *E. coli* DH5α (ATCC 53868), were purchased from the China Center of Industrial Culture Collection (CICC). The identity of the bacteria was confirmed based on strain authentication by the supplier, morphological characteristics, and selective culturing on LB agar. Initially, preparations were made for both liquid and agar culture media. Beef extract, peptone, and sodium chloride were added to the conical flask, after which 1000 mL deionized water was added, stirred with a glass rod, and all was to be dissolved in water to make up the liquid culture. The pH was adjusted to between 7.2 and 7.6 using 10 g/hg hydrochloric acid or 10 g/hg sodium hydroxide. To evaluate antibacterial properties, culture media, along with other consumables such as Petri dishes, centrifuge tubes, saline solution, and Erlenmeyer flasks, were sterilized at 121 °C for one hour to ensure complete sterility. The sterilized culture media were then poured into Petri dishes until one-third full. After solidification, three specimens of each composite were UV-sterilized for three hours and each was placed in 10 mL of liquid medium in individual flasks. These were then incubated at 37 °C with agitation and light for 24 h. Post-incubation, the samples were shaken in saline within centrifuge tubes, diluted sequentially in five 10-fold steps, and plated on solid media. Following another 24-h incubation at 37 °C with light, colonies were counted to assess antibacterial effectiveness, with results averaged over three replicates.

### 3.16 Fruit preservation test

To assess the fruit preservation ability of MPBS/CNT-ZNP nanocomposite films, bananas were used as a test subject due to their well-documented sensitivity to microbial spoilage and rapid physical changes during storage, making them a widely accepted model fruit in preservation studies. Fresh, unripe bananas of uniform size (approximately 6 ± 1 cm in length and 2.5 ± 0.2 cm in diameter) and weight (95 ± 10 g) were selected from the same batch to minimize variability. The bananas were purchased from a local farmers' market in Zigong, Sichuan Province, China, and were sourced from Fushun County, Zigong. Before coating, all bananas were carefully washed with distilled water to remove surface contaminants and then dried at room temperature for 30 min. Four groups were prepared: (1) uncoated (control), (2) coated with pure PBS film, (3) coated with MPBS film, and (4) coated with MPBS/CNT-ZNP_0.1 g/hg nanocomposite film. Each banana was wrapped in its respective film type and stored at room temperature (28 ± 2 °C) and 60 % relative humidity. Each group consisted of three film-coated samples, with three bananas individually wrapped in each type of film to obtain the average values for weight loss, total soluble solids (TSS, °Brix), and pH. Weight loss (%) was determined by recording the initial and final weights of the bananas. The total soluble solids (TSS) value was measured using a digital refractometer (model JBM-20, Shenzhen FNIRSI Technology Co., Ltd., Shenzhen, China). The pH value was determined using a pH meter (model PH-100 A, Shanghai Xuancheng Instrument Co., Ltd., Shanghai, China). To better track surface color changes during storage, photographs were taken at fixed time points under consistent lighting. RGB values were extracted from representative areas on the banana peel using ImageJ software (version 1.53 t, National Institutes of Health, Bethesda, MD, USA). These values were used to monitor visual ripening and browning over time, and the results are presented in Table S12. This provided a straightforward, quantitative supplement to visual observations.

### 3.17 Meat shelf-life test

To evaluate the shelf-life extension capabilities of the MPBS/CNT-ZNP nanocomposite films, fresh chicken samples were selected as test materials. Fresh chicken samples were selected as test materials. The skinless chicken breast samples were purchased from a local supermarket in Zigong, Sichuan Province, China, on the same day of testing. Four groups were prepared: (1) uncoated (control), (2) wrapped in pure PBS film, (3) wrapped in MPBS film, and (4) wrapped in MPBS/CNT-ZNP_0.1 g/hg nanocomposite film. The chicken samples used were skinless breast meat, which is known for its uniform composition and is commonly used in packaging studies. Each piece used in testing weighed approximately 1 g, consistent across all sample groups. Each sample was stored at 4 °C to simulate refrigerated conditions, and bacterial counts were measured at 36-h intervals up to 108 h. Each group consisted of three film-coated samples, with three pieces of chicken individually wrapped in each type of film to obtain average microbial growth values. Samples were swabbed, and bacteria were cultured on nutrient agar plates to assess microbial proliferation.

### 3.18 Statistical analysis

All experimental data were obtained from at least three independent replicates and expressed as mean ± standard deviation. One-way ANOVA followed by post-hoc Tukey's test was used to determine significant differences between groups. The significance levels are indicated as *P* ≤ 0.05, *P* ≤ 0.01, and *P* ≤ 0.001.

## 4 Results and discussion

### 4.1 Chemical analysis for PBS and MPBS

**Fig. 1:**
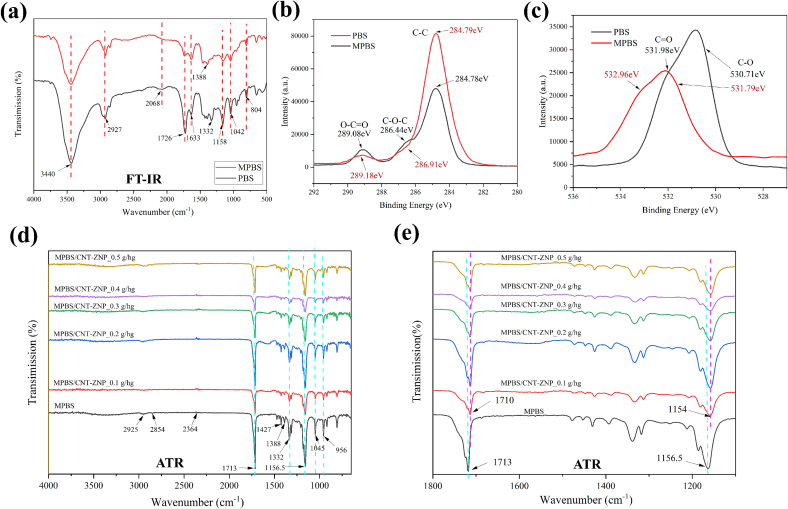
(a) FTIR spectra of PBS and MPBS; (b) XPS (C1s) spectra of PBS and MPBS; (c) XPS (O1s) spectra of PBS and MPBS; (d) ATR-FTIR spectra of MPBS and MPBS/CNT-ZNP composites; (e) Enlarged view of selected ATR peaks for MPBS/CNT-ZNP composites.

Fig. 1a shows the FTIR spectra for PBS and MPBS, revealing characteristic peaks at 3440 cm^−1^, 2927 cm^−1^, 1726 cm^−1^, and 1633 cm^−1^, corresponding to OH, C—H, and C=O groups in the ester structure of both polymers (Cai et al., 2013). In MPBS, the intensity of the 1726 cm^−1^ peak decreases while the 1633 cm^−1^ peak increases, suggesting successful grafting of MAH onto PBS. This likely occurs via anhydride esterification with OH or C=O-OH in PBS, resulting in new C=O in the anhydride group and a reduction in OH peak intensity at 3440 cm^−1^.

XPS analysis, as shown in Fig. 1b and c, further confirms modification by MAH. The C1s spectrum of MPBS shows a > 60 % increase in C—C bonding intensity compared to PBS, likely due to additional C—C linkages facilitated by MAH. C-O-C and O-C=O peaks in MPBS are slightly lower, which may indicate chain scission or rearrangement during grafting (Greczynski et al., 2021; Isaacs et al., 2021). The O1s spectrum also displays reduced C=O and C—O peak intensities in MPBS, likely due to MAH modifying the chemical environment of oxygen-containing groups and potential chain scission. Moreover, higher binding energy shifts in these peaks indicate an altered electronic environment due to anhydride groups from MAH (Klyushin et al., 2014). The esterification process might reduce original carboxylic groups, further lowering C=O intensity. Overall, these FTIR and XPS results validate significant structural modifications in MPBS, highlighting the impact of MAH grafting on the polymer's properties and supporting its enhanced performance.

### 4.2 Chemical analysis for MPBS and MPBS/CNT-ZNP nanocomposites

ATR-FTIR spectroscopy was used to analyze MPBS nanocomposites with varying CNT-ZNP contents. Fig. 1d and e show that the main infrared peaks in MPBS/CNT-ZNP nanocomposites correspond to those in PBS. Key peaks include C—H stretching in methylene groups at 2925 cm^−1^ and 2854 cm^−1^ and a strong carbonyl peak around 2364 cm^−1^. Peaks associated with -CH₃ groups are seen at 1427, 1388, 1332, and 1313 cm^−1^, while peaks at 1713 cm^−1^ and 1156.5 cm^−1^ (C=O and C-O-C in ester bonds) shift rightward after CNT-ZNP addition. This shift suggests interactions involving coordination bonds and possible hydrogen bonding between MPBS and CNT-ZNP (Cai et al., 2019; Fornaro et al., 2015; Uesaka et al., 2000; Younis et al., 2021).

**Fig. 2:**
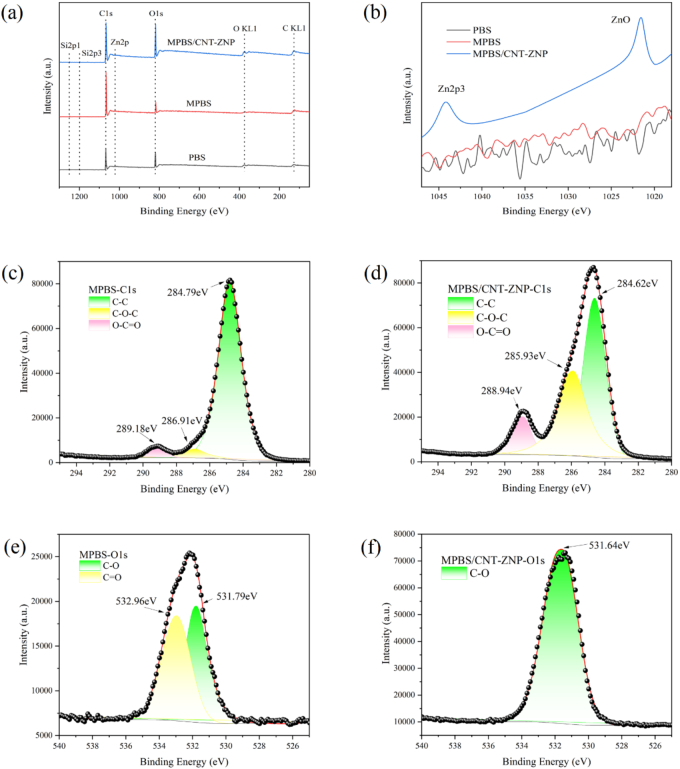
XPS analysis (a) full scan spectrum of PBS, MPBS and MPBS/CNT-ZNP nanocomposite (b) narrow scan of PBS, MPBS and MPBS/CNT-ZNP nanocomposite (c) C1s for MPBS (d) C1s for MPBS/CNT-ZNP (e) O1s for MPBS (f) O1s for MPBS/CNT-ZNP.

To further investigate these effects, XPS analysis was conducted (Fig. 2). A notable increase in the C1s peak intensity in MPBS compared to PBS aligns with previous findings (Fig. 2b). The O1s peak intensity is also higher in MPBS/CNT-ZNP composites, likely due to the oxygen atoms in ZnO from CNT-ZNP. Despite the low 0.1 g/hg CNT-ZNP concentration, ZnO presence in MPBS is confirmed by peaks around 1022 eV and 1044 eV (Fig. 2b). C1s and O1s spectra (Figs. 2c-2f) show a rightward shift (indicating lower binding energy) for O-C-O and O-C=O peaks, attributed to interactions with Zn ions from CNT-ZNP. This binding energy reduction likely results from coordination with less electronegative atoms like Zn ions, increasing electron density (Greczynski et al., 2021; Shao et al., 2024). Additional electron density from hydrogen bonding may further lower binding energy, while reduced density would increase it (Kerber et al., 1996). These shifts indicate hydrogen bonding interactions between ZnO in CNT-ZNP and MPBS, suggesting enhanced compatibility and structural integrity in the nanocomposite.

### 4.3 Gel permeation chromatography analysis

**Fig. 3:**
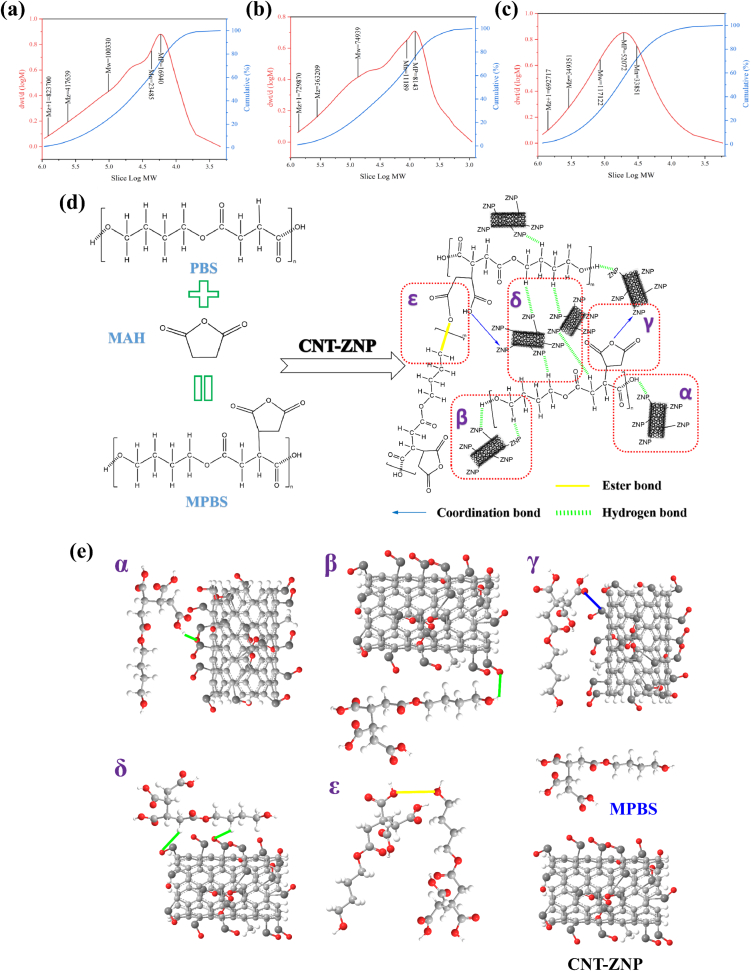
GPC analysis of (a) PBS (b) MPBS (c) MPBS/CNT-ZNP nanocomposite; (d) and (e) Chemical reaction between MPBS and CNT-ZNP.

Gel permeation chromatography (GPC) was used to analyze the molecular weight distribution of PBS, MPBS, and MPBS with 0.1 g/hg CNT-ZNP nanocomposites (Fig. 3). The GPC profile for pure PBS showed a high molecular weight (Mw) of 100,330 Da and a broad distribution (Mz + 1 of 823,700). Grafting maleic anhydride onto PBS reduced Mw to 74,939 Da, indicating possible chain cleavage during modification. This trend was also seen in Mn, suggesting a uniform grafting effect across polymer chains (Yoshikawa & Tsubokawa, 1996). Similar reductions in Mw during MAH grafting have been reported by Krause-Sammartino et al., who observed that the use of dicumyl peroxide (DCP) as an initiator in the grafting of maleic anhydride onto polypropylene led to chain scission and a consequent decrease in molecular weight (Krause Sammartino et al., 2006; Montanheiro et al., 2016). With 0.1 g/hg CNT-ZNP, Mw increased to 117,122 Da, possibly due to enhanced polymer chain interactions facilitated by CNT-ZNP, which promotes intermolecular bonding. The increase in Mp and molecular weight fractions also indicates that CNT-ZNP may act as a compatibilizer within the polymer matrix (Cheng et al., 2016; Oliveira et al., 2018). Furthermore, Mz and Mz + 1 for MPBS/CNT-ZNP_0.1 g/hg were lower than in pure PBS but higher than MPBS alone, suggesting that CNT-ZNP encourages a more even distribution of higher molecular weight fractions. These changes in molecular weight and distribution reflect strong chemical and physical interactions at the molecular level, affecting the composite's properties.

Additional FTIR, XPS, and GPC analyses suggest a complex 3D cross-linked network structure in MPBS/CNT-ZNP composites. Fig. 3d illustrates five reaction mechanisms that contribute to this structure: (α) Zn^+^ ions from CNT-ZNP interact with –COOH groups in MPBS, initiating bonding; (β) Zn^+^ ions bond with –OH groups, adding structural complexity; (γ) Zn^+^ reacts with anhydride groups, deepening integration; (δ) ZnO forms hydrogen bonds with –CH groups, enhancing flexibility; and (ε) anhydride groups form ester bonds with –OH or –COOH groups in PBS, increasing stability. These interactions collectively create a 3D network that improves material properties, marking a significant advancement in nanocomposite development.

### 4.4 Mechanical properties

**Fig. 4:**
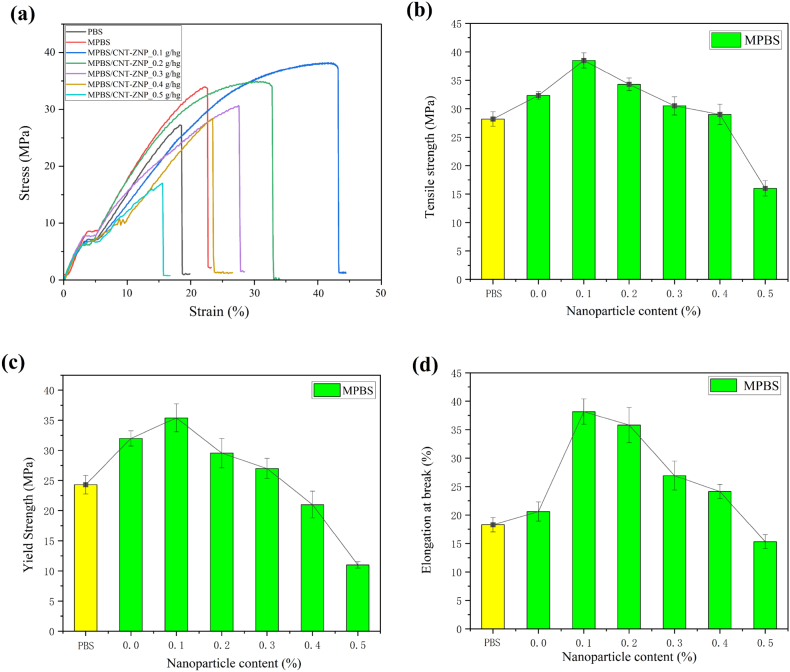
MPBS/CNT-ZNP of (a) stress-strain curve (b) tensile strength (c) yield strength (d) elongation at break.

Fig. 4 presents the impact of varying CNT-ZNP content on the tensile properties of MPBS/CNT-ZNP composites. Pure PBS showed a tensile strength of 28.2 MPa. With MAH and CNT-ZNP additions, tensile strength initially increased, reaching a peak of 38.5 MPa (a 36.5 % improvement) at 0.1 g/hg CNT-ZNP before declining at higher concentrations due to nanomaterial agglomeration and reduced matrix bonding capacity (Fig. 4b) (Deshmukh et al., 2014). Elongation at break followed a similar trend, with 0.1 g/hg CNT-ZNP showing the highest increase at 108.9 g/hg over PBS, consistent with enhanced tensile strength (Fig. 4d). Above 0.1 g/hg, the uneven distribution led to increased brittleness and a decline in mechanical performance due to agglomeration and incompatibility (Wen et al., 2020). These findings demonstrate that 0.1 g/hg CNT-ZNP optimally enhances tensile strength and toughness, leveraging effective interfacial bonding and a synergistic effect between MAH and CNT-ZNP. Compared to previously reported PBS nanocomposites containing CNT, the developed composite demonstrates superior mechanical performance, achieving a 36.5 % increase in tensile strength and a 108 % increase in elongation at break with only 0.1 g/hg filler. In most CNT-based PBS systems reported in the literature, ≥1 wt% loading is typically required to obtain similar or even lower enhancements (Liu et al., 2023; Luo et al., 2022; De Guzman et al., 2020), underscoring the high efficiency of the current formulation. Notably, this performance value is significantly higher than that of PBS-Bionolle #1020MD produced by Showa Highpolymer Co., Ltd. (See Table S1).

The improvements in mechanical properties were statistically significant compared to pure PBS. For instance, the tensile strength and elongation at break of the 0.1 g/hg CNT-ZNP composite showed *P* ≤ 0.001, indicating a highly significant enhancement. Similarly, the yield strength of MPBS and MPBS/CNT-ZNP composites exhibited *P* ≤ 0.01 or better, confirming the effectiveness of the modifications. However, at higher CNT-ZNP concentrations (e.g., 0.5 g/hg), the mechanical properties deteriorated significantly (P ≤ 0.001), highlighting the detrimental effects of agglomeration and poor dispersion.

### 4.5 Fracture surface morphology and EDS

**Fig. 5:**
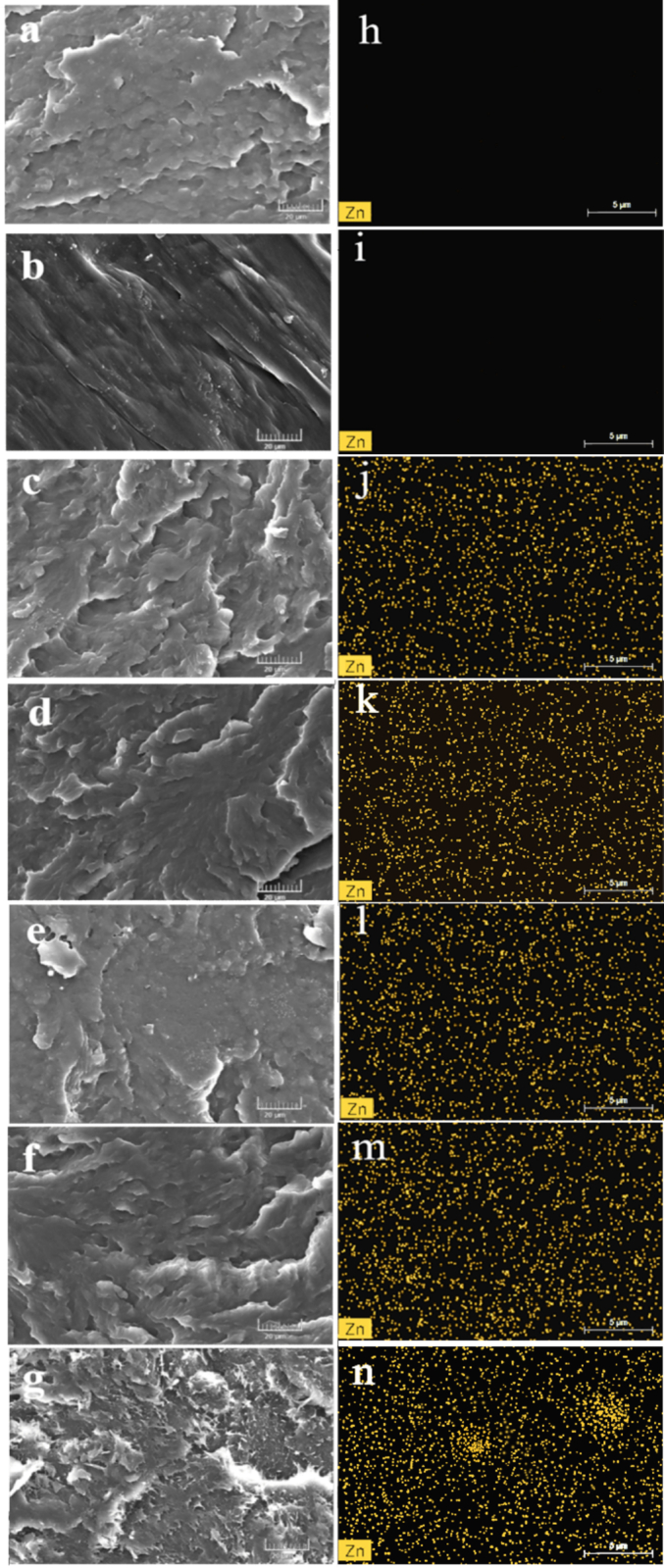
The SEM morphology of composite materials of (a) PBS (b) MPBS (c) MPBS/CNT-ZNP_0.1 g/hg (d)MPBS/CNT-ZNP_0.2 g/hg (e) MPBS/CNT-ZNP_0.3 g/hg (f)MPBS/ CNT-ZNP_0.4 g/hg (g)MPBS/CNT-ZNP_0.5 g/hg. EDS diagram of composite of (h) PBS (i) MPBS (j) MPBS/CNT-ZNP_0.1 g/hg (k) MPBS/CNT-ZNP_0.2 g/hg (l) MPBS/CNT-ZNP_0.3 g/hg (m) MPBS/ CNT-ZNP_0.4 g/hg (n) MPBS/CNT-ZNP_0.5 g/hg.

Fig. 5 shows the fracture cross-sections morphology of MPBS/CNT-ZNP composites. Pure PBS (Fig. 5a) has a smooth, flat surface. With the addition of 0.1 g/hg CNT-ZNP, the MPBS surface adopts a fish-scale morphology with evenly distributed fillers (Fig. 5c), indicating good interfacial bonding and dispersion from MAH modification. At higher CNT-ZNP content (0.5 g/hg, Fig. 5g), voids and particle aggregation appear, negatively affecting tensile strength and elongation (Tsou et al., 2019; Tsou, Chen, et al., 2022; Wu et al., 2019). EDS analysis (Figs. 5h-5n) confirms Zn distribution, showing no Zn in pure PBS or MPBS but uniform dispersion in composites with 0.1 g/hg CNT-ZNP (Fig. 5j). At 0.2 g/hg CNT-ZNP, minor agglomeration begins, leading to internal defects that reduce tensile performance, with further aggregation at higher concentrations (Figs. 5l-5n). This suggests that 0.1 g/hg CNT-ZNP optimally enhances mechanical strength and toughness through effective dispersion.

### 4.6 Comprehensive thermal analysis

**Fig. 6:**
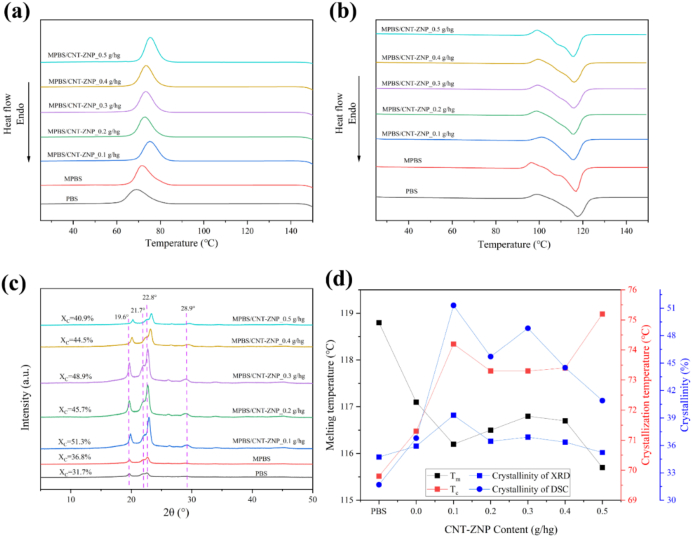
The DSC values of composites of (a) first cooling (b) second heating (c) XRD curves of composite materials (d) all data for DSC and XRD.

Fig. 6 and Table S4 present the differential scanning calorimetry (DSC) results of PBS, MPBS, and MPBS/CNT-ZNP nanocomposites, evaluating crystallization temperature (Tc), melting temperature (Tm), and crystallinity (Xc). The incorporation of MAH in MPBS resulted in a slight increase in Tc and Xc compared to neat PBS, likely due to the anhydride groups promoting more organized molecular alignment.

The introduction of CNT-ZNP further increased Tc, indicating enhanced crystallization kinetics. At a CNT-ZNP concentration of 0.1 g/hg, the MPBS/CNT-ZNP composite exhibited the highest Tc, enthalpy of fusion, and crystallinity. These enhancements can be attributed to the uniform dispersion of CNT-ZNP within the polymer matrix, which provides additional nucleation sites and promotes stable crystal growth (Tsou, Ma, et al., 2022). The maximum Xc value reached 39.30 % at 0.1 g/hg CNT-ZNP. However, further increases in CNT-ZNP content led to a decrease in crystallinity, likely due to nanoparticle agglomeration disrupting the distribution of nucleation sites (Choi et al., 2022).

The Tm values showed only minor variations across the samples, with the lowest Tm observed at 0.1 g/hg CNT-ZNP. This may be due to the formation of smaller and less thermally stable crystallites induced by the nanofillers (Righetti et al., 2023). Overall, the results suggest that 0.1 g/hg CNT-ZNP is the optimal loading level, effectively enhancing crystallization behavior while maintaining a balance between thermal stability and structural integrity.

Previous studies incorporating nanofibrillated cellulose, surface-treated coffee husk, and gel-grafted lignin into PBS also reported increases in crystallinity and Tc (Lule et al., 2022; Platnieks et al., 2021; Zhou et al., 2021). However, these fillers were typically added at concentrations greater than 1 g/hg, highlighting the superior nucleation efficiency of CNT-ZNP at much lower dosages.

### 4.7 X-ray diffraction

X-ray diffraction (XRD) was performed to investigate the crystallization behavior of PBS and MPBS/CNT-ZNP composites. Fig. 6c displays the diffraction peaks of PBS at 2θ values of 19.6°, 21.7°, 22.8°, and 28.9°, which are indicative of a monoclinic crystal structure, consistent with previous findings (Wen et al., 2021). These results confirm that the incorporation of maleic anhydride (MAH) into PBS increases its crystallinity. Furthermore, the addition of CNT-ZNP significantly enhances crystallization, with the highest crystallinity observed at a CNT-ZNP concentration of 0.1 g/hg.

In MPBS/CNT-ZNP composites, the XRD patterns show slight shifts in diffraction peaks, accompanied by an increase in peak intensity, which suggests a reduction in lattice spacing and improved dispersion of the nanofillers. Additionally, the appearance of new peaks at 26.1° and 28.9° provides evidence of structural changes, likely due to the interaction between CNT-ZNP and the PBS matrix. These peaks may correspond to the characteristic diffraction peaks of ZNP and CNT (Kumar et al., 2024). The XRD analysis reveals that the addition of CNT-ZNP enhances both the crystallization and structural compatibility within the composite. These structural modifications contribute to the improved mechanical and thermal properties observed in the MPBS/CNT-ZNP composites.

### 4.8 Thermostability

Fig. S3 and Table S5 present the thermogravimetric analysis (TGA) results for PBS, MPBS, and MPBS/CNT-ZNP nanocomposites. The onset decomposition temperatures, including T5 % (5 % mass loss), T10 % (10 % mass loss), and the maximum degradation temperature (Tmax), all increased upon incorporation of CNT-ZNP, with the best thermal stability observed at 0.1 g/hg CNT-ZNP. Specifically, MPBS/CNT-ZNP_0.1 g/hg showed a T5% of 351.0 °C and Tmax of 427.8 °C, significantly higher than those of pure PBS (T5% = 331.9 °C, Tmax = 425.2 °C). The results suggest that CNT-ZNP acts as a thermal insulator, limiting heat transfer and stabilizing the polymer chains.

Additionally, MPBS exhibited higher thermal resistance than PBS alone, which can be attributed to the chemical grafting of MAH. The anhydride groups may form covalent or strong polar interactions with the CNT-ZNP surface, restricting polymer chain mobility and enhancing thermal stability. These interfacial effects likely lead to tighter molecular packing and delayed thermal degradation.

### 4.9 Water vapor barrier performance

**Fig. 7:**
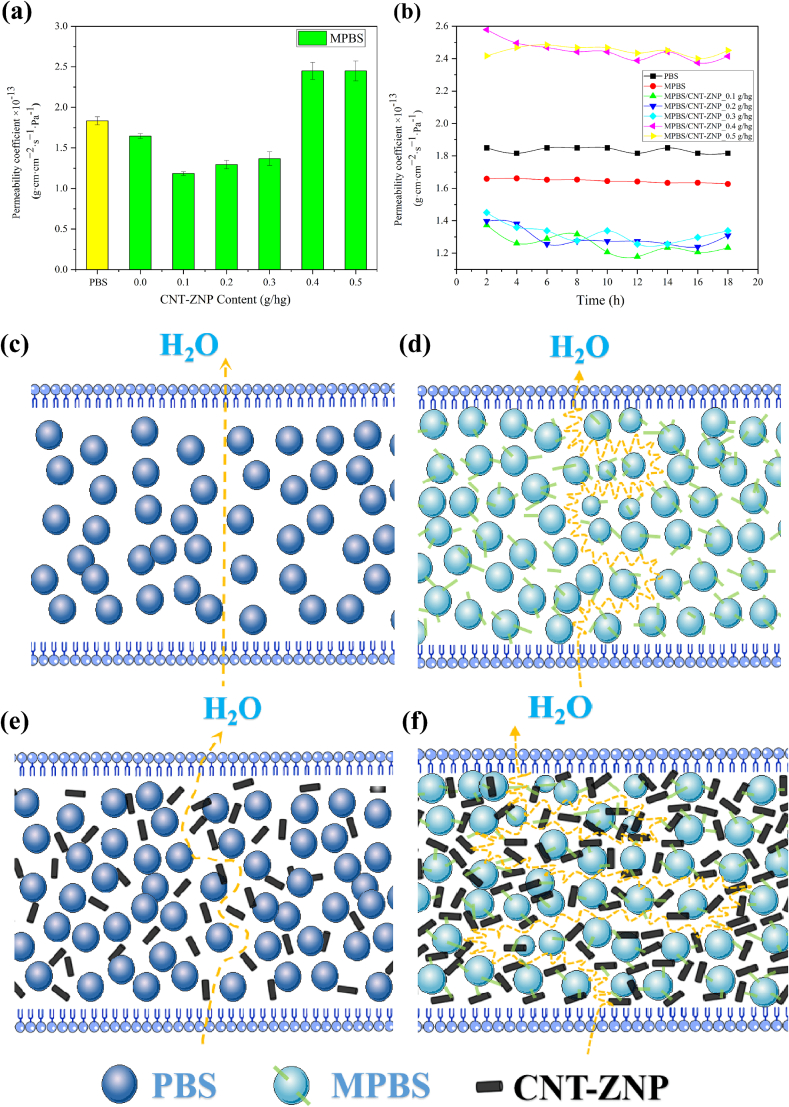
Water vapor barrier properties of (a) average water vapor permeability of PBS, MPBS/CNT-ZNP nanocomposite (b) PBS, MPBS/CNT-ZNP composite material real time moisture permeability coefficient. Schematic diagram of H_2_O molecules diffusing through PBS, MPBS and MPBS/CNT-ZNP nanocomposites: (c) diffusion path through PBS (d) diffusion path through MPBS (e) diffusion path around PBS/CNT-ZNP nanocomposite (f) narrower diffusion path around MPBS/CNT-ZNP nanocomposites.

Fig. 7 and Table S6 show the water vapor permeability coefficient (WVPC) for CNT-ZNP/MPBS nanocomposites at 26 °C and 90 % humidity. Initially, WVPC decreases with nanomaterial content, reaching the lowest value at 0.1 g/hg CNT-ZNP (1.18 × 10^−13^ g·cm/cm^2^·s·Pa), indicating optimal barrier properties. This improvement is due to the complex path created by nanomaterials, which hinders water vapor transmission. At higher CNT-ZNP levels (0.4 g/hg and 0.5 g/hg), WVPC increases due to microagglomeration, forming defects that ease water molecule passage. Even so, WVPC for modified MPBS with CNT-ZNP remains lower than unmodified PBS, suggesting tighter binding and improved compatibility. Similar trends have been reported in previous work. For example, Ge et al. (Ge et al., 2024) incorporated ZnO-coated multi-walled carbon nanotubes into a PBS matrix at a loading of 0.1 phr and achieved a minimum WVPC of 1.26 × 10^−13^ g·cm/cm^2^·s·Pa. In comparison, the present study achieved an even lower WVPC of 1.185 × 10^−13^ g·cm/cm^2^·s·Pa at only 0.1 g/hg CNT-ZNP loading, demonstrating superior barrier performance. This improvement is likely attributed to the enhanced interfacial interactions between MPBS and CNT-ZNP, as well as the more compact microstructure facilitated by the anhydride modification of PBS.

The improvements in water vapor barrier properties were statistically significant compared to pure PBS (Table S6). The 0.1 g/hg CNT-ZNP composite showed the most significant reduction in WVPC (*P* ≤ 0.001), highlighting the effectiveness of the nanomaterial dispersion and interfacial interactions. The MPBS sample also exhibited a statistically significant improvement (*P* ≤ 0.05) over PBS, confirming the role of MAH in enhancing barrier properties. At higher CNT-ZNP concentrations (0.4 g/hg and 0.5 g/hg), the WVPC values increased significantly (P ≤ 0.05), reflecting the detrimental effects of agglomeration and reduced compatibility.

As shown in Fig. 7c-f, water molecules follow a straightforward path in unmodified PBS (Fig. 7c). The addition of MAH in MPBS alters this path, tightening the molecular chain packing and reducing permeability (Fig. 7d). In MPBS/CNT-ZNP composites, water molecules move in a zigzag pattern around CNT-ZNP (Fig. 7e), with MAH-Zn^+^ interactions further complicating the path, requiring water molecules to travel longer distances to permeate (Fig. 7f).

### 4.10 Water absorption and contact angle

Fig. S4a and S4b show water absorption results for the nanocomposites over 24 and 48 h. PBS had higher water absorption than the MPBS/CNT-ZNP composites, indicating that MAH-modified composites have lower water uptake due to better compatibility and bonding within the matrix. The lowest water absorption rate was observed at 0.1 g/hg CNT-ZNP content, suggesting a compact structure with improved tensile strength, crystallinity, and thermal resistance. Higher nanomaterial content led to increased water absorption due to agglomeration and the formation of gaps in the matrix (Tsou, Zeng, Tsou, et al., 2022). Fig. S4c and S4d illustrate contact angle results, showing optimal hydrophobicity at 0.1 g/hg CNT-ZNP. This is attributed to interactions between nanomaterials and hydroxyl groups, enhancing hydrophobic properties through tighter internal bonding. The MAH-modified MPBS matrix promotes reactions between MPBS and Zn^+^ in CNT-ZNP, reducing hydroxyl groups and further increasing hydrophobicity, consistent with the water absorption results and supporting improved tensile performance at 0.1 g/hg nanofiller content.

### 4.11 Biodegradability

Fig. S5 and Table S7 show the weight loss rates of PBS and MPBS/CNT-ZNP nanocomposites after 180 days of soil burial. All samples exhibited increasing weight loss over time, with a steady rise up to 120 days, followed by a notable acceleration. This “self-acceleration” effect indicates that after 120 days, the materials further decompose, leading to higher degradation rates. The MPBS/CNT-ZNP nanocomposites with 0.1 g/hg CNT-ZNP showed the lowest weight loss, attributed to the uniform dispersion of nanofillers and strong compatibility with the polymer matrix, forming a dense structure that limits microbial and moisture infiltration. This structure contributes to the optimal tensile properties seen at this concentration. Higher degradation rates in MPBS/CNT-ZNP composites with increased CNT-ZNP content may stem from matrix degradation or the hydrophilic nature of MPBS, which enhances susceptibility to microbial or moisture-induced breakdown in soil.

### 4.12 Quantitative antibacterial tests

Fig. S6a and S6b shows quantitative test results of analyzing *E. coli* in MPBS/CNT-ZNP nanocomposites. Compared to Pure PBS material, MPBS modified with MAH has shown a minor impact on *E. coli.* Previous research has indicated that MAH possesses some antibacterial properties (Du et al., 2025). The introduction of CNT-ZNP nanomaterials led to a significant reduction in the number of bacterial colonies in the nanocomposite materials, indicating that the antibacterial effect becomes increasingly pronounced with higher concentrations of CNT-ZNP nanomaterials. Notably, when the concentration of added CNT-ZNP nanomaterials reached 0.5 g/hg, the sterilization rate surpassed 99 %. This finding highlights the potent antimicrobial properties of CNT-ZNP nanomaterials in enhancing the effectiveness of MPBS in combating bacterial growth.

The antibacterial efficacy of the nanocomposites was statistically significant compared to pure PBS (see Table S8). As shown in the quantitative results, MPBS exhibited a moderate but statistically significant reduction in bacterial counts compared to PBS (*P* ≤ 0.05). The MPBS/CNT-ZNP composites demonstrated highly significant reductions in bacterial counts at all concentrations, with *P* ≤ 0.001 for 0.1 g/hg, 0.2 g/hg, 0.3 g/hg, 0.4 g/hg, and 0.5 g/hg. The most pronounced effect was observed at 0.5 g/hg, where bacterial counts were reduced to nearly zero (2 CFU, P ≤ 0.001), highlighting the potent antimicrobial properties of CNT-ZNP nanomaterials in enhancing the effectiveness of MPBS in combating bacterial growth.

It has been demonstrated that ZNP nanoparticles within nanocomposites are highly effective in killing *E. coli* (Swamiappan et al., 2018). This efficacy is attributed to the release of Zn^2+^ ions, which can penetrate bacterial cells, disrupt membrane integrity, and subsequently inactivate the bacteria (Dutta et al., 2013). Additionally, the generation of reactive oxygen species (ROS) by ZNP nanoparticles contributes significantly to their antibacterial properties (Pauzi et al., 2020). In the present work, the MPBS and MPBS/CNT-ZNP nanocomposites exhibited marginally enhanced antibacterial effectiveness compared to neat PBS. This enhancement may be attributed to two primary factors. First, the presence of anhydride groups in MPBS potentially increases surface acidity or disrupts microbial membranes, consistent with the findings reported by Tsou et al. (Tsou, Zeng, et al., 2022), where maleic anhydride-grafted polyesters showed moderate antibacterial activity. Second, a synergistic effect is likely to occur when ZNP and CNT coexist, as supported by the study of Mohamed et al. (Mohamed et al., 2025), which reported enhanced antibacterial activity in systems combining ZnO nanoparticles with carbon-based nanomaterials, due to intensified ROS generation and improved bacterial surface contact. These mechanisms, together with previous literature, provide a reasonable explanation for the observed improvement in antibacterial performance of the MPBS/CNT-ZNP nanocomposites.

### 4.13 Anti-inflammatory test

The anti-inflammatory test evaluated the effects of PBS, MPBS, and MPBS/CNT-ZNP nanocomposites on protein denaturation, a critical factor in preserving food quality. Fig. S6c shows that PBS had no effect on protein denaturation, while MPBS exhibited minimal inhibition. However, the MPBS/CNT-ZNP nanocomposite significantly inhibited protein denaturation, indicating strong anti-inflammatory properties. This suggests that the CNT-ZNP nanocomposite can effectively reduce protein degradation, thereby helping to maintain the nutritional value, texture, and taste of food products. Its application in food packaging could slow degradation processes, extending the freshness and shelf life of food items.

### 4.14 Fruit preservation

**Fig. 8:**
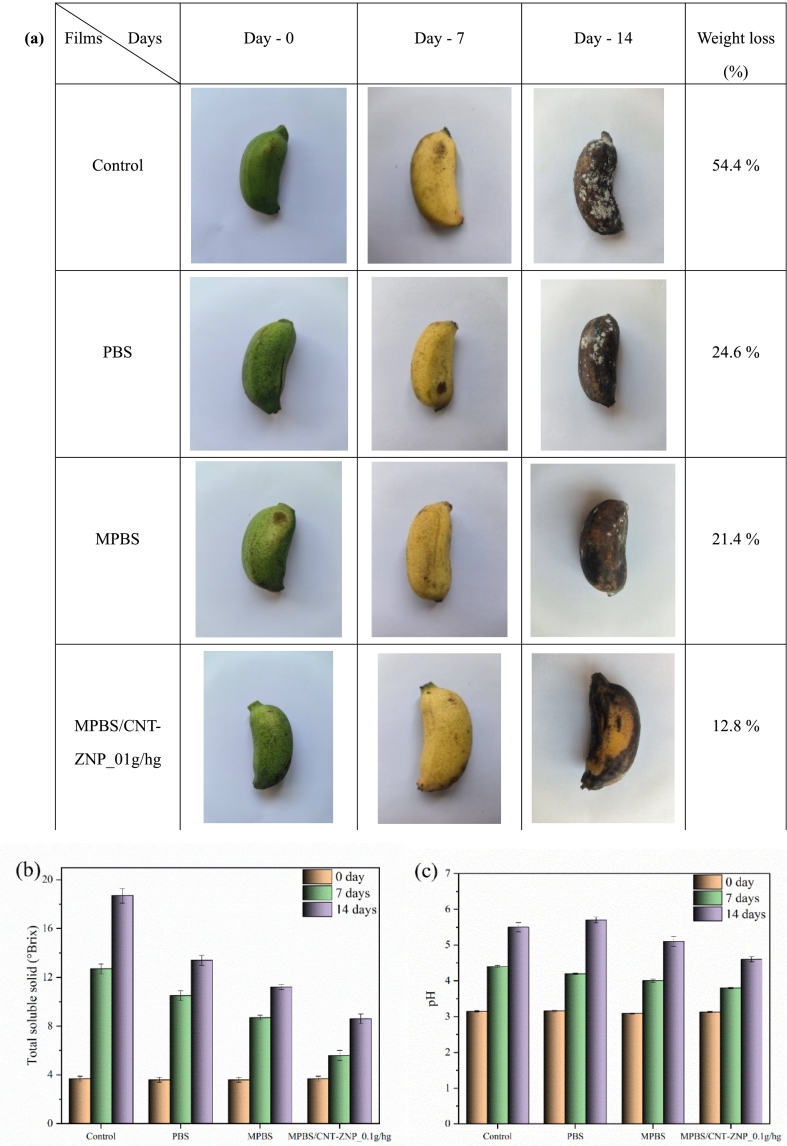
(a) Images showing the color changes and mold formation on bananas over time; (b) total soluble solids; and (c) pH of bananas coated with different film materials on Days 0, 7, and 14.

This section evaluates the preservation efficacy of MPBS/CNT-ZNP nanocomposite films on bananas over 14 days. Fig. 8a shows images of bananas coated with different films on Days 0, 7, and 14, along with weight loss measurements after 14 days. Initially, all bananas were green (Day 0). By Day 7, bananas in all groups ripened to yellow, indicating normal ripening. However, by Day 14, clear differences emerged. Uncoated bananas and those coated with pure PBS had darkened significantly, showing a brownish-black hue with visible white mold, suggesting microbial growth, and weight loss exceeded 50 %. In contrast, bananas coated with MPBS film showed less discoloration and reduced mold formation, likely due to the mild antimicrobial properties of anhydride groups in MPBS (Haktaniyan et al., 2022), which may inhibit microbial cell membranes. The MPBS/CNT-ZNP_0.1 g/hg nanocomposite film demonstrated the best preservation effect: bananas retained a fresh yellow color, minimal mold, and the lowest recorded weight loss at 12.8 %. This enhanced preservation effect is attributed to the nanocomposite film's superior antibacterial, antifungal, and barrier properties, which effectively inhibit microbial growth and prevent moisture loss, thereby extending the freshness of the fruit.

Total soluble solids (TSS), measured in °Brix, is another key indicator of fruit ripening. As shown in Fig. 8b and Table S9, the TSS of uncoated bananas (Control) increased from 3.7 ± 0.2°Brix on Day 0 to 18.7 ± 0.6°Brix on Day 14, reflecting significant sugar accumulation and ripening. The substantial weight loss observed in the Control group, exceeding 50 %, also contributed to the higher TSS values, as water loss concentrates sugars and other soluble solids (Alabi et al., 2022). Bananas coated with PBS showed a slower increase in TSS, reaching 13.4 ± 0.4°Brix by Day 14 (*P* ≤ 0.001), which can be partially attributed to reduced water loss compared to the Control. MPBS-coated bananas exhibited even better preservation, with TSS increasing to only 11.2 ± 0.2°Brix by Day 14 (P ≤ 0.001), further highlighting the role of reduced moisture loss in maintaining fruit quality. The MPBS/CNT-ZNP_0.1 g/hg nanocomposite film demonstrated the best performance, with TSS increasing to just 8.6 ± 0.4°Brix by Day 14 (P ≤ 0.001). This significant reduction in TSS accumulation further confirms the nanocomposite's ability to delay ripening, minimize water loss, and maintain fruit quality (Alabi et al., 2022; Pasha et al., 2023).

The pH of banana pulp is a critical indicator of ripening and preservation efficacy. As shown in Fig. 8c Table S9, the pH of uncoated bananas (Control) increased significantly from 3.15 ± 0.02 on Day 0 to 5.5 ± 0.13 on Day 14, reflecting advanced ripening and spoilage. Bananas coated with PBS showed a similar trend, though the pH increase was slightly slower (5.7 ± 0.08 on Day 14, *P* ≤ 0.05). The pH increase in uncoated bananas on Day 14 was less pronounced compared to PBS-coated bananas. This phenomenon may be attributed to the fact that uncoated bananas, after 14 days, experienced spoilage, leading to a lower pH compared to the ripening stage due to the production of organic acids and acidic metabolites by microbial decomposition (Perumal et al., 2021; Zhao & Ndayambaje et al., 2022). MPBS-coated bananas exhibited a more moderate pH increase, reaching 5.1 ± 0.14 by Day 14 (*P* ≤ 0.01), indicating better preservation. The MPBS/CNT-ZNP_0.1 g/hg nanocomposite film demonstrated the most effective preservation, with the pH increasing only to 4.6 ± 0.07 by Day 14 (P ≤ 0.001). This significant delay in pH change highlights the nanocomposite's ability to slow ripening and microbial activity (Abdul Malek et al., 2025; Ghimire et al., 2021). The MPBS/CNT-ZNP_0.1 g/hg nanocomposite film exhibited significant efficacy in preserving the postharvest quality of bananas. This was evidenced by reduced pH fluctuations, lower TSS accumulation, minimal mold proliferation, and notably decreased weight loss over a 14-day storage period. Such outcomes suggest that the nanocomposite film effectively mitigates microbial spoilage and moisture loss, which are critical factors influencing fruit ripening and deterioration. These findings align with previous studies where nanocomposite coatings, particularly those incorporating ZnO and Ag₂O nanoparticles, have demonstrated enhanced barrier and antimicrobial properties, leading to improved preservation of banana quality during storage (Aruwajoye et al., 2025). Similarly, the incorporation of halloysite nanotubes into packaging materials has been shown to delay ripening and senescence in bananas by stabilizing pH and TSS levels (Kumar et al., 2024). Furthermore, chitosan-based coatings enriched with nanoclay have been reported to retard ripening processes, as indicated by lower TSS accumulation and weight loss (Roidoung et al., 2025). Collectively, these studies underscore the potential of nanocomposite films in extending the shelf life and maintaining the quality of perishable fruits like bananas.

To quantitatively evaluate the visual color changes in bananas during storage, RGB values were extracted using ImageJ software and summarized in Table S12. On Day 0, all banana samples exhibited green tones, with relatively low R (red) values (Control: 120.0; PBS: 124.7; MPBS: 129.8; MPBS/CNT-ZNP: 130.6), and high G and B values, indicating unripe fruit. By Day 7, all groups transitioned to yellow hues, as reflected by a sharp increase in R values (≥170) and a decline in G and B channels, consistent with natural ripening. However, by Day 14, color differences became more evident. The uncoated bananas (Control) and those coated with PBS showed the highest R values (179.1 and 172.8, respectively) and elevated G and B values (≥173), suggesting excessive ripening and browning. The MPBS-coated bananas had slightly lower R and B values, suggesting reduced browning and better surface preservation. Notably, the MPBS/CNT-ZNP_0.1 g/hg group exhibited the lowest R (171.0), G (168.6), and B (164.7) values on Day 14, indicating more stable color retention with less browning and decay. This quantitative color analysis is consistent with the visual observations presented in Fig. 8a and reinforces the conclusion that MPBS/CNT-ZNP nanocomposite films offer better preservation performance. The delayed ripening and browning observed in the coated bananas can likely be attributed to the improved barrier properties of the film, which limit oxygen and moisture transmission, as well as the antimicrobial effects of the nanofillers that reduce microbial-induced spoilage. Similar effects have been reported in previous studies using Zn -based edible coatings, which were shown to slow enzymatic browning and preserve fruit color during storage by minimizing oxidative stress and microbial growth (Horison et al., 2022). These findings together support the idea that the synergistic action of ZNP and CNT in the composite film plays a key role in extending the visual freshness of the fruit.

### 4.15 Shelf life of chicken

**Fig. 9:**
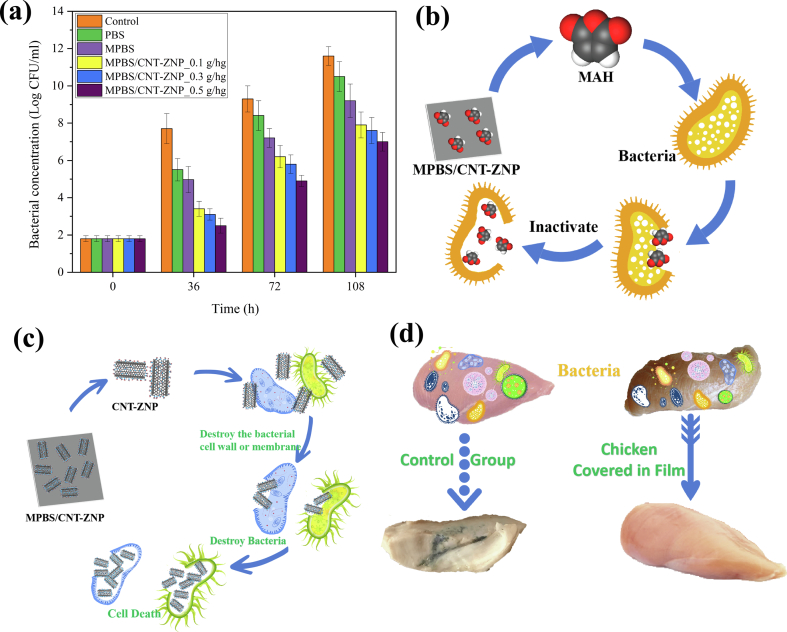
Comparative Analysis of Chicken Shelf-Life Preservation of (a) bacterial counts on raw chicken measured at 36, 72, and 108 h for control, PBS, MPBS, and MPBS/CNT-ZNP nanocomposite films (b) the MAH functional group's antibacterial properties in the nanocomposite films (c) CNT-ZNP disrupting bacterial cell walls (d) barrier effects of the films against water vapor and bacterial ingress.

Bacterial counts on raw chicken were tracked at 36, 72, and 108 h for control, PBS- wrapped, MPBS-wrapped, and MPBS/CNT-ZNP-wrapped samples (Fig. 9a; Table S10). Using 8 Log CFU mL^−1^ as a commonly cited spoilage threshold for chilled poultry, the control exceeded the limit by 72 h (9.3) and reached 11.6 by 108 h. Wrapping with PBS delayed growth only at the early stage (5.5 at 36 h, P = 0.014 vs control), but counts were not significantly different from control at 76 h (8.4, P = 0.194) and remained high at 108 h (10.5, P = 0.098). MPBS provided a stronger delay, keeping counts below the threshold at 72 h (7.2, P = 0.005) and reaching 9.2 at 108 h (P = 0.006). Incorporation of CNT-ZNP produced a clear, dose-dependent improvement. At 0.1 g/hg, counts were 3.4 (P < 0.001) at 36 h and 6.2 (P < 0.001) at 72 h, crossing the threshold only by 108 h (8.3, P < 0.001). At 0.3 g/hg and 0.5 g/hg, counts remained below 8 Log CFU mL^−1^ through 108 h (7.6 and 7.0, respectively; both P < 0.001 vs control at all timepoints), indicating an extension of the microbiological shelf life to at least 108 h under refrigeration.

The superior antimicrobial effect of MPBS/CNT-ZNP films is attributed to the inherent antibacterial properties of MAH (Fig. 9b), along with the disruptive action of CNT-ZNP on bacterial cell walls, which effectively leads to bacterial death (Fig. 9c). Additionally, these films serve as a barrier, reducing water vapor ingress and blocking bacterial entry from the environment (Hu et al., 2018) (Fig. 9d). These results highlight MPBS/CNT-ZNP nanocomposites' effectiveness in extending chicken shelf life, demonstrating their potential as advanced food packaging materials that enhance both freshness and safety.

## 5 Conclusion

In this study, MPBS/CNT-ZNP nanocomposites were successfully developed using ZnO-coated carbon nanotubes as a reinforcing agent. A small amount of CNT-ZNP (0.1 g/hg) significantly improved the mechanical, thermal, and barrier properties of PBS, while also imparting strong antibacterial and preservation functions. Structural and thermal analyses confirmed enhanced crystallinity and thermal stability, and the composites exhibited excellent moisture resistance and slower degradation under soil burial. Practical food preservation tests demonstrated that the nanocomposite film effectively reduced microbial spoilage in chicken and delayed ripening in bananas, as reflected in lower weight loss, reduced pH changes, and TSS accumulation. These results highlight the potential of MPBS/CNT-ZNP composites as multifunctional materials with low filler requirements for sustainable food packaging.

## CRediT authorship contribution statement

**Chi-Hui Tsou:** Writing – review & editing, Methodology, Investigation, Funding acquisition, Formal analysis, Conceptualization. **Xin Huang:** Writing – original draft, Project administration, Investigation, Formal analysis, Data curation. **Fei-Fan Ge:** Writing – original draft, Validation, Software, Formal analysis, Data curation. **Jarrn-Horng Lin:** Resources, Investigation, Formal analysis. **Pranut Potiyaraj:** Writing – review & editing, Supervision, Resources, Formal analysis. **Charasphat Preuksarattanawut:** Writing – review & editing, Resources, Investigation, Conceptualization. **Tao Yang:** Visualization, Investigation. **Xue-Fei Hu:** Project administration, Investigation.

## Declaration of competing interest

The authors declare that they have no known competing financial interests or personal relationships that could have appeared to influence the work reported in this paper.

## Data Availability

Data will be made available on request.

## Appendix A Supplementary data

Supplementary material providing additional experimental details, Figures S1-S6, and Table S1-S12. Image 1
