# Supplementary material for: Enhanced performance of nanoplate-carbon nanotube reinforced poly(butylene succinate) nanocomposites for sustainable packaging and multifunctional applications
Source: Food Chem X. 2025 Sep 9;31:102989. doi: 10.1016/j.fochx.2025.102989 (PMC12475868; doi:10.1016/j.fochx.2025.102989)
Supplement: Supplementary file 1 — Supplementary material providing additional experimental details, Figures S1-S6, and Table S1-S12. [file mmc1.docx]

**Supplementary materials**

Table S1. Comparative molecular and mechanical properties of selected commercial PBS materials. The TH-803S sample was used in this study, while Bionolle #1001MD and #1020MD are included for literature-based comparison. Data such as molecular weight (Mw), melt flow index (MFI), density, tensile strength, and elongation at break were compiled from published sources.

| Type of PBS | Mw (g/mol) | MFI  (g/10 min) | Density (g/cm³) | Tensile Strength (MPa) | Elongation at Break (%) | Reference |
| --- | --- | --- | --- | --- | --- | --- |
| TH-803S | 1.3 × 10⁵ | 10–29.4 | 1.26 | 28.2 | 18.29 | This work; Bing et al., 2024 |
| Bionolle #1001MD | 2.23 × 10⁵ | 1.5 | 1.26 | 41.95 ± 0.63 | 468 ± 25 | Xie et al., 2014; Ostrowsk et al., 2019 |
| Bionolle #1020MD | 1.4 × 10⁵ | 25 | 1.23 | 32.6 ± 2.7 | 10.9 ± 2.6 | Phua et al., 2013 |

Table S2. Raw material content

| Sample | PBS (g/hg) | PBS-g-MAH (g/hg) | CNT-ZNP (g/hg) |
| --- | --- | --- | --- |
| PBS | 100 | 0 | 0 |
| MPBS | 90 | 10 | 0 |
| MPBS/CNT-ZNP_0.1 g/hg | 89.91 | 9.99 | 0.1 |
| MPBS/CNT-ZNP_0.2 g/hg | 89.82 | 9.98 | 0.2 |
| MPBS/CNT-ZNP_0.3 g/hg | 89.73 | 9.97 | 0.3 |
| MPBS/CNT-ZNP_0.4 g/hg | 89.64 | 9.96 | 0.4 |
| MPBS/CNT-ZNP_0.5 g/hg | 89.55 | 9.95 | 0.5 |

Table S3. Mechanical properties of PBS and MPBS/CNT-ZNP composite material

| Sample | Tensile strength (MPa) | Yield strength (MPa) | Elongation at break (%) |
| --- | --- | --- | --- |
| PBS | 28.20±1.26 | 24.30±1.56 | 18.29±1.16 |
| MPBS | 32.33 ± 0.68 (P ≤ 0.05) | 32.00 ± 1.25 (P ≤ 0.01) | 20.60 ± 1.48 (P ≤ 0.05) |
| MPBS/CNT-ZNP_0.1 g/hg | 38.50 ± 1.35 (P ≤ 0.001) | 35.40 ± 2.34 (P ≤ 0.001) | 38.16 ± 2.23 (P ≤ 0.001) |
| MPBS/CNT-ZNP_0.2 g/hg | 34.28 ± 1.11 (P ≤ 0.01) | 29.54 ± 2.44 (P ≤ 0.05) | 35.80 ± 3.14 (P ≤ 0.001) |
| MPBS/CNT-ZNP_0.3 g/hg | 30.50 ± 1.59 (P ≤ 0.05) | 27.00 ± 1.69 (P ≤ 0.05) | 26.92 ± 2.55 (P ≤ 0.01) |
| MPBS/CNT-ZNP_0.4 g/hg | 29.00 ± 1.78 (P ≤ 0.05) | 21.00 ± 2.22 (P ≤ 0.05) | 24.11 ± 1.25 (P ≤ 0.05) |
| MPBS/CNT-ZNP_0.5 g/hg | 16.00 ± 1.35 (P ≤ 0.001) | 11.00 ± 0.53 (P ≤ 0.001) | 15.32 ± 1.22 (P ≤ 0.001) |

Table S4. Differential scanning calorimetry analysis of PBS and MPBS/CNT-ZNP composites

| Sample | *Tc* (℃) | *ΔHc* (J/g) | *Tm* (℃) | *ΔHm* (J/g) | *Xc* (%) |
| --- | --- | --- | --- | --- | --- |
| PBS | 69.8 | 75.31 | 118.8 | 69.45 | 34.73 |
| MPBS | 71.3 | 76.59 | 117.1 | 71.83 | 35.92 |
| MPBS/CNT-ZNP_0.1 g/hg | 74.2 | 78.01 | 116.2 | 78.52 | 39.30 |
| MPBS/CNT-ZNP_0.2 g/hg | 73.3 | 73.79 | 116.5 | 72.76 | 36.45 |
| MPBS/CNT-ZNP_0.3 g/hg | 73.3 | 77.3 | 116.8 | 73.6 | 36.91 |
| MPBS/CNT-ZNP_0.4 g/hg | 73.4 | 77.14 | 116.7 | 72.43 | 36.36 |
| MPBS/CNT-ZNP_0.5 g/hg | 75.2 | 75.7 | 115.7 | 70.12 | 35.23 |

Table S5. Thermogravimetric data of PBS and MPBS/CNT-ZNP composites

| Sample | *T_5 %_* ( ℃) | *T_10%_* ( ℃) | *T_max_* ( ℃) | *T_DTG_* ( ℃) | Residual quantity (%) |
| --- | --- | --- | --- | --- | --- |
| PBS | 331.90 | 368.79 | 425.21 | 397.85 | 14.28 |
| MPBS | 338.07 | 369.78 | 424.11 | 402.53 | 15.76 |
| MPBS/CNT-ZNP_0.1 g/hg | 351.03 | 379.74 | 427.82 | 406.64 | 22.85 |
| MPBS/CNT-ZNP_0.2 g/hg | 347.71 | 378.10 | 426.06 | 404.46 | 24.87 |
| MPBS/CNT-ZNP_0.3 g/hg | 339.92 | 377.18 | 425.01 | 403.03 | 18.78 |
| MPBS/CNT-ZNP_0.4 g/hg | 341.98 | 377.88 | 425.86 | 404.13 | 19.44 |
| MPBS/CNT-ZNP_0.5 g/hg | 340.68 | 368.53 | 417.61 | 399.96 | 29.33 |

Table S6. Water vapor barrier properties of average moisture permeability of PBS, MPBS/CNT-ZNP composite materials

| Sample | Permeability coefficient ×10^-13^ (g·cm·cm^−2^·s^−1^·Pa^-1^) |
| --- | --- |
| PBS | 1.834 ± 0.053 |
| MPBS | 1.645 ± 0.031 (P ≤ 0.05) |
| MPBS/CNT-ZNP_0.1 g/hg | 1.185 ± 0.021 (P ≤ 0.001) |
| MPBS/CNT-ZNP_0.2 g/hg | 1.295 ± 0.052 (P ≤ 0.01) |
| MPBS/CNT-ZNP_0.3 g/hg | 1.368 ± 0.084 (P ≤ 0.05) |
| MPBS/CNT-ZNP_0.4 g/hg | 2.449 ± 0.106 (P ≤ 0.05) |
| MPBS/CNT-ZNP_0.5 g/hg | 2.452 ± 0.123 (P ≤ 0.05) |

Table S7. Degradation performance of PBS, MPBS, and MPBS/CNT-ZNP nanocomposites

| Sample | 30 days (%) | 60 days (%) | 90 days (%) | 120 days (%) | 150 days (%) | 180 days (%) |
| --- | --- | --- | --- | --- | --- | --- |
| PBS | 2.54±0.34 | 11.93±1.23 | 16.44±1.75 | 22.89±2.45 | 37.33±2.54 | 45.79±3.45 |
| MPBS | 5.38±0.32 | 14.27±1.54 | 21.31±1.86 | 26.42±2.64 | 47.72±2.67 | 57.68±3.45 |
| MPBS/CNT-ZNP_0.1 g/hg | 5.24±0.23 | 11.79±1.45 | 18.83±1.66 | 24.97±2.36 | 45.00±2.74 | 52.85±3.54 |
| MPBS/CNT-ZNP_0.2 g/hg | 5.42±0.23 | 12.63±1.47 | 21.22±1.96 | 26.71±2.86 | 49.05±2.45 | 54.42±3.54 |
| MPBS/CNT-ZNP_0.3 g/hg | 5.82±0.54 | 12.81±1.63 | 20.11±1.86 | 26.77±2.65 | 49.79±2.64 | 54.12±3.56 |
| MPBS/CNT-ZNP_0.4 g/hg | 5.43±0.34 | 14.03±1.46 | 22.44±1.68 | 31.51±2.74 | 54.02±2.34 | 64.59±3.64 |
| MPBS/CNT-ZNP_0.5 g/hg | 5.54±0.56 | 13.38±1.67 | 23.82±1.76 | 33.59±2.85 | 58.57±2.34 | 65.30±3.35 |

Table S8. Number of *E. coli* resistant colonies and Antibacterial rate

| Sample | 10^6^ CFU | P-value | Antibacterial rate (%) |
| --- | --- | --- | --- |
| PBS | 864 ± 22 | - | 0 |
| MPBS | 783 ± 24 | P ≤ 0.05 | 9.38±0.23 |
| MPBS/CNT-ZNP_0.1 g/hg | 128 ± 11 | P ≤ 0.001 | 85.20±3.4 |
| MPBS/CNT-ZNP_0.2 g/hg | 74 ± 8 | P ≤ 0.001 | 91.43±2.3 |
| MPBS/CNT-ZNP_0.3 g/hg | 35 ± 4 | P ≤ 0.001 | 95.95±1.3 |
| MPBS/CNT-ZNP_0.4 g/hg | 10 ± 2 | P ≤ 0.001 | 98.84±0.6 |
| MPBS/CNT-ZNP_0.5 g/hg | 2 ± 0 | P ≤ 0.001 | 99.77±0.3 |

Table S9. Effect of PBS, MPBS and MPBS/CNT-ZNP composite films on the pulp pH of banana over a storage period of 14 days at ambient condition.

| Sample | pH | | |
| --- | --- | --- | --- |
|  | 0 day | 7 days | 14 days |
| Control | 3.15 ± 0.02 | 4.4 ± 0.03 | 5.5 ± 0.13 |
| PBS | 3.16 ± 0.02 | 4.2 ± 0.02 (P ≤ 0.05) | 5.7 ± 0.08 (P ≤ 0.05) |
| MPBS | 3.09 ± 0.01 | 4.0 ± 0.04 (P ≤ 0.01) | 5.1 ± 0.14 (P ≤ 0.01) |
| MPBS/CNT-ZNP_0.1 g/hg | 3.13 ± 0.02 | 3.8 ± 0.02 (P ≤ 0.001) | 4.6 ± 0.07 (P ≤ 0.001) |

Table S10. Comparative analysis of chicken shelf-Life preservation of bacterial counts on raw chicken at 36-hour intervals comparing control, pure PBS films, and MPBS/CNT-ZNP nanocomposites

| Sample | 36h (Log CFU/ml) | 72h (Log CFU/ml) | 108h (Log CFU/ml) |
| --- | --- | --- | --- |
| Control | 7.7 ± 0.8 | 9.3 ± 0.7 | 11.6 ± 0.5 |
| PBS | 5.5± 0.6 (P = 0.014) | 8.4 ± 0.8 (P = 0.194) | 10.5 ± 0.8 (P = 0.098) |
| MPBS | 4.97 ± 0.7 (P = 0.006) | 7.2 ± 0.5 (P = 0.005) | 9.2 ± 0.9 (P = 0.006) |
| MPBS/CNT-ZNP_0.1 g/hg | 3.4 ± 0.4 (P < 0.001) | 6.2 ± 0.6 (P < 0.001) | 7.9 ± 0.7 (P < 0.001) |
| MPBS/CNT-ZNP_0.3 g/hg | 3.1 ± 0.3 (P < 0.001) | 5.8 ± 0.5 (P < 0.001) | 7.6 ± 0.7 (P < 0.001) |
| MPBS/CNT-ZNP_0.5 g/hg | 2.5 ± 0.4 (P < 0.001) | 4.9 ± 0.3 (P < 0.001) | 7.0 ± 0.5 (P < 0.001) |

Table S11. Effect of PBS, MPBS and MPBS/CNT-ZNP composite films on the Total soluble solid of banana over a storage period of 14 days at ambient condition.

| Sample | Total soluble solid (°Brix) | | |
| --- | --- | --- | --- |
|  | 0 day | 7 days | 14 days |
| Control | 3.7 ± 0.2 | 12.7 ± 0.4 | 18.7 ± 0.6 |
| PBS | 3.6 ± 0.2 | 10.5 ± 0.4 (P ≤ 0.01) | 13.4 ± 0.4 (P ≤ 0.001) |
| MPBS | 3.6 ± 0.2 | 8.7 ± 0.2 (P ≤ 0.001) | 11.2 ± 0.2 (P ≤ 0.001) |
| MPBS/CNT-ZNP_0.1 g/hg | 3.7 ± 0.2 | 5.6 ± 0.4 (P ≤ 0.001) | 8.6 ± 0.4 (P ≤ 0.001) |

Table S12. Effect of PBS, MPBS, and MPBS/CNT-ZNP composite films on the color of bananas measured by image analysis using ImageJ software over a 14-day storage period at ambient conditions.

| Samples | 0 day | | | 7 days | | | 14 days | | |
| --- | --- | --- | --- | --- | --- | --- | --- | --- | --- |
|  | R | G | B | R | G | B | R | G | B |
| Control | 120.0 | 132.8 | 131.6 | 173.5 | 169.0 | 165.5 | 179.1 | 180.6 | 180.7 |
| PBS | 124.7 | 136.5 | 130 | 174.8 | 171.2 | 168.5 | 172.8 | 173.4 | 173.6 |
| MPBS | 129.8 | 139.7 | 125.6 | 171.7 | 167.2 | 167.7 | 173.1 | 174.7 | 173.0 |
| MPBS/CNT-ZNP_0.1 g/hg | 130.6 | 143.4 | 139.0 | 170.6 | 164.1 | 156.5 | 171.0 | 168.6 | 164.7 |


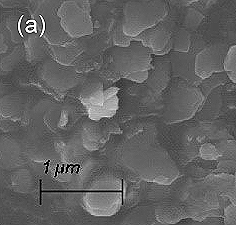

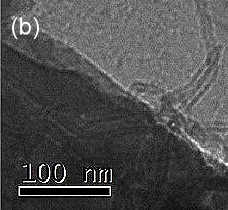


Figure S1. (a) SEM image (b) TEM image


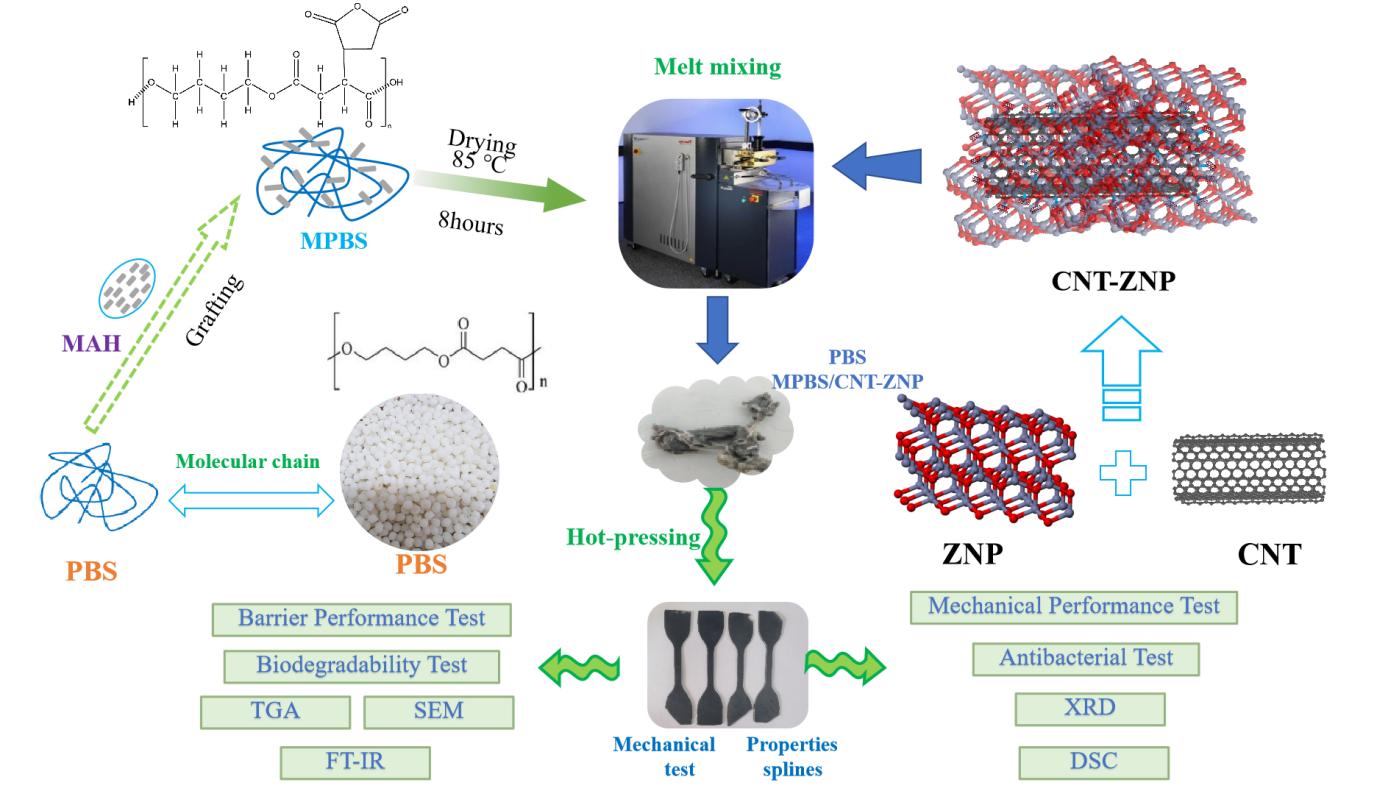


Figure S2. Schematic of fabricating PBS and MPBS/CNT-ZNP nanocomposite


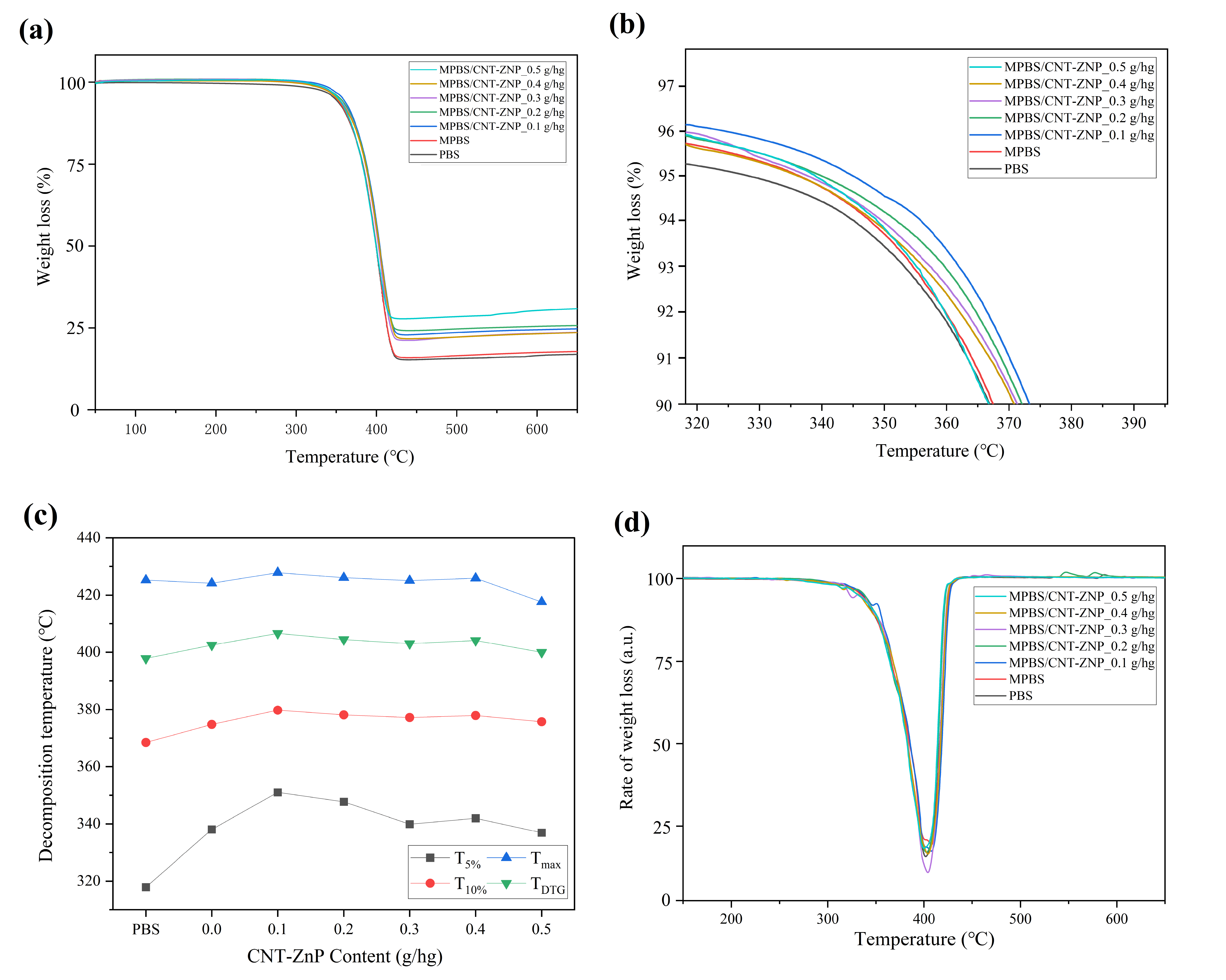


Figure S3. Thermogravimetric spectra of all samples (a) TGA curves (b) partial enlarged image of TGA curves (c) data for TGA curves (d) DTG curves


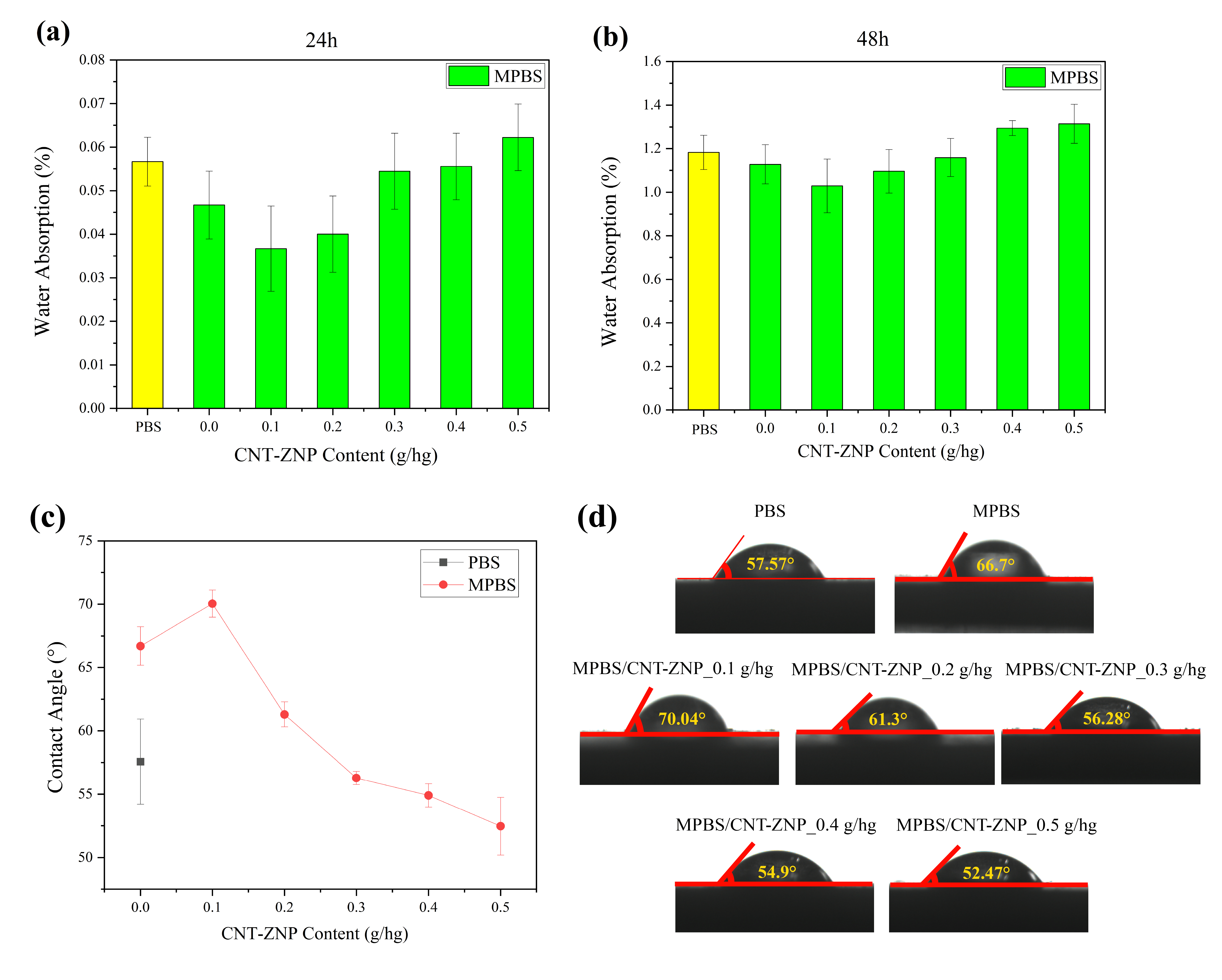


Figure S4. Water absorptions of (a) 24h (b) 48h (c) Contact angle data (d) Contact angle image


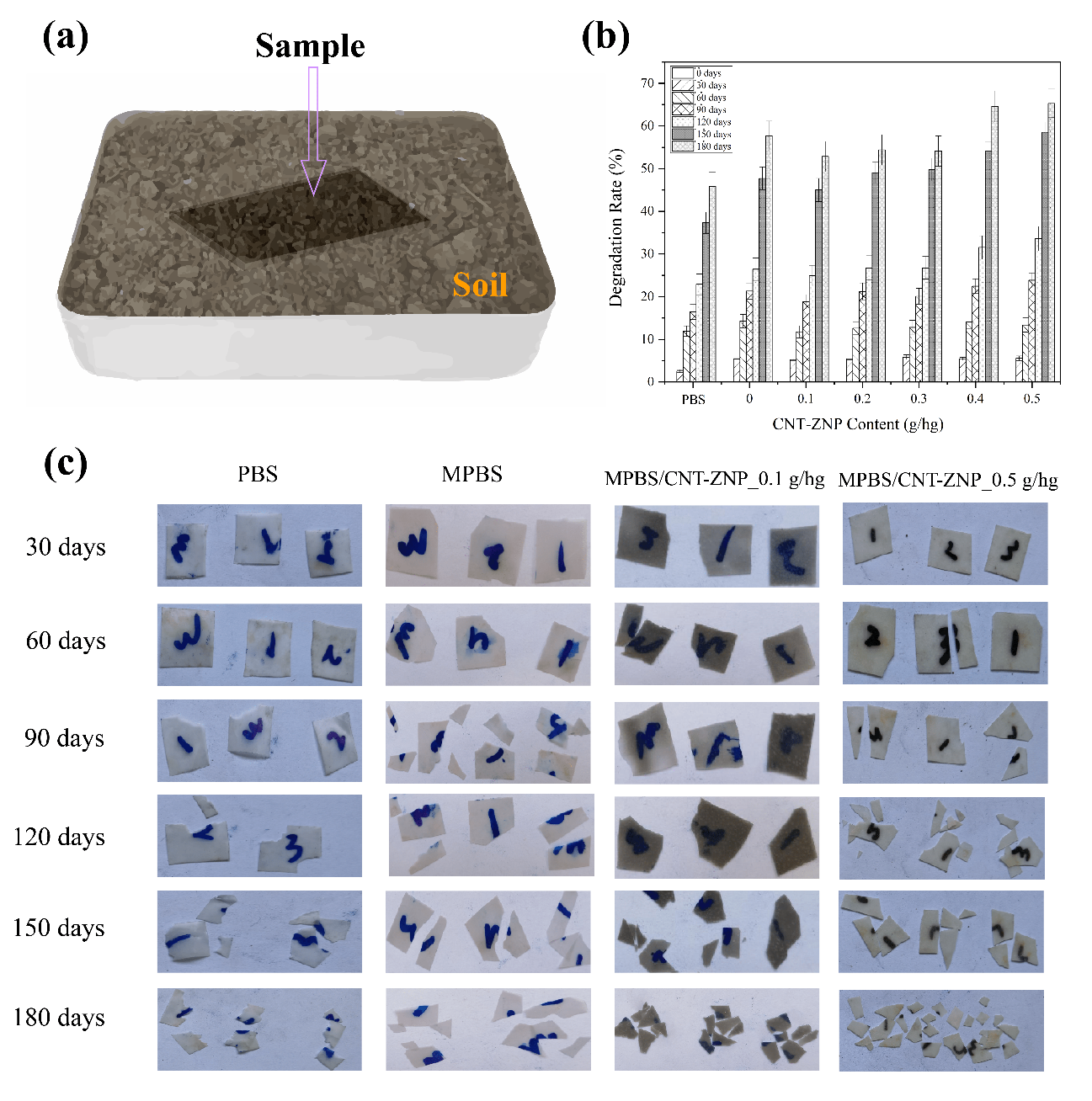


Figure S5. Degradation performance of PBS, MPBS, and MPBS/CNT-ZNP nanocomposites: (a) Schematic diagram of buried soil test; (b) Degradation weight loss rate; (c) Samples with different burial times.


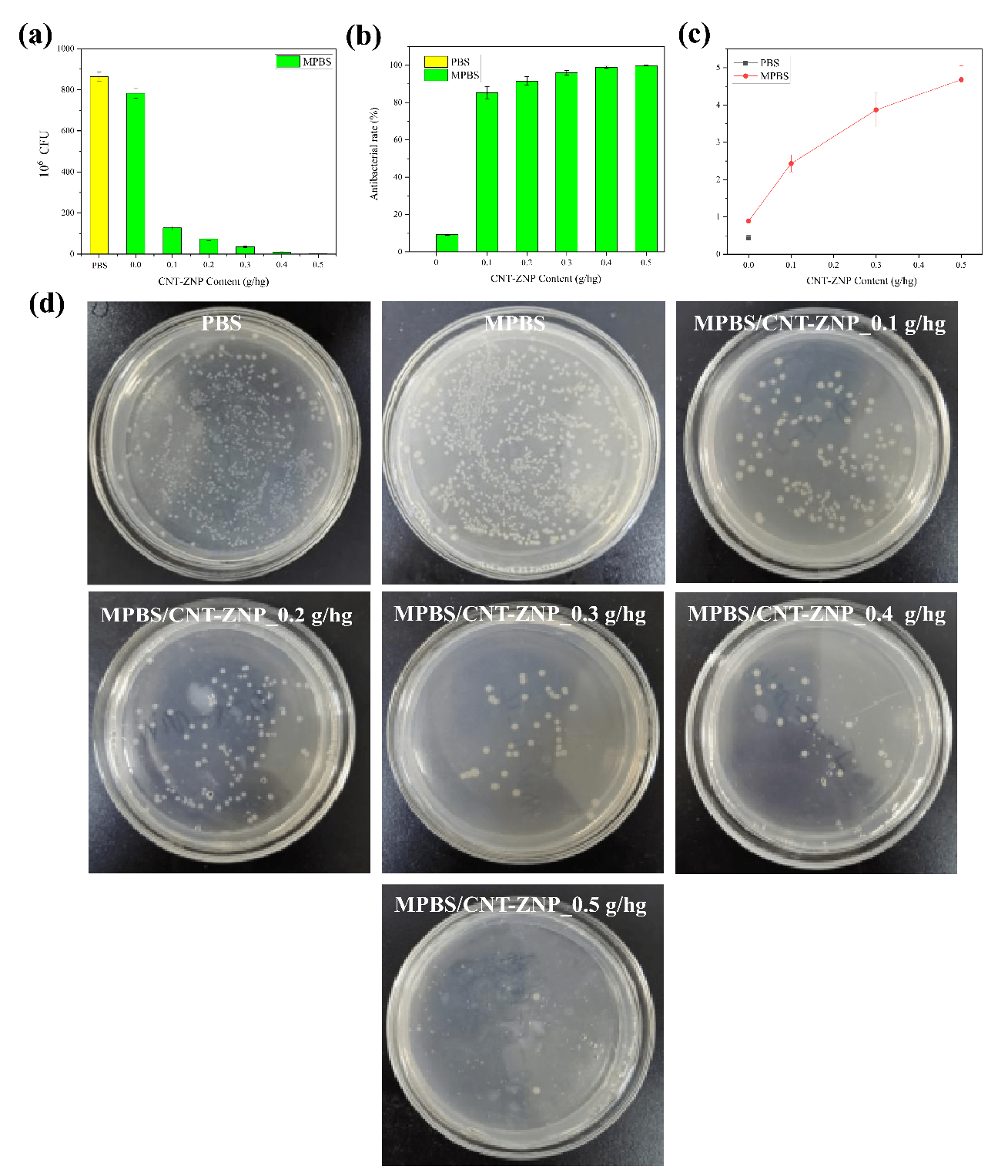


Figure S6. (a) *E. coli* resistant colonies (b) Antibacterial rate (c) Anti-inflammatory properties (d) Images for Number of *E. coli* resistant colonies in samples
